# Phyllotaxis, ontogeny and CT imaging: old and new approaches to understanding optimal seed packing in Middle Jurassic *Araucaria mirabilis* cones

**DOI:** 10.1093/aob/mcaf325

**Published:** 2025-12-26

**Authors:** Mariah M Howell, Ronny Rößler, Carole T Gee

**Affiliations:** Bonn Institute for Organismic Biology, Division of Paleontology, University of Bonn, Nussallee 8, Bonn 53115, Germany; Museum für Naturkunde Chemnitz, Moritzstraße 20, Chemnitz 09111, Germany; Bonn Institute for Organismic Biology, Division of Paleontology, University of Bonn, Nussallee 8, Bonn 53115, Germany

**Keywords:** *Araucaria mirabilis*, Araucariaceae, seed cone phyllotaxis, micro-computed tomography, seed packing, Middle Jurassic, Bosques Petrificados de Jaramillo National Park, Cerro Cuadrado Petrified Forest, ontogenetic spiral, cone seediness

## Abstract

**Background and Aims:**

Fibonacci spiral phyllotaxis is overwhelmingly the most common leaf arrangement among plants, both today and in the fossil record. Spiral phyllotaxis appeared as early as the Late Devonian, some 380 Mya. This mathematical property has been studied rigorously in living conifer species; however, the phyllotaxis of fossil conifer seed cones remains under-studied, including that of the Middle Jurassic seed cones of *Araucaria mirabilis*.

**Methods:**

Twenty-one *A. mirabilis* seed cones from the Bosques Petrificados de Jaramillo National Park, Argentina, were analysed for their morphometrics, phyllotaxis and seediness. For each cone, the number of seeds in the cone, in addition to the number of clockwise and anticlockwise parastichies, was counted on the well-preserved cone surface. Micro-CT was used to observe the internal arrangement of the seeds in the cone in a non-destructive manner. Then, Avizo was used to visualize one clockwise and one anticlockwise parastichy from each cone to determine the number of seeds in the spiral and the angle of rotation of the spiral.

**Key Results:**

Here, we show that clockwise ontogenetic chirality and greater axis length and width are positively correlated with greater seediness in *A. mirabilis* seed cones. Cones with clockwise ontogenetic chirality have moderately tighter spirals (*P* < 0.05, *r* = 0.2) with more seeds in them (*P* < 0.05, *r* = 0.3). Likewise, a taller axis is associated with a greater circumference (*P* < 0.01, *r* = 0.6), which, in turn, is correlated with more bract/scale complexes (*P* < 0.05, *r* = 0.05). The most common phyllotaxis is 13,21 (64 %), with 360° spirals (48 %) and clockwise ontogenetic chirality (66 %).

**Conclusions:**

Visualizing internal morphometrics and seed arrangements show which characteristics contribute to optimal seed packing. Thus, micro-CT imaging, in addition to traditional methods, enables a deeper study of conifer cone morphology and construction.

## INTRODUCTION

Today, Fibonacci spirals constitute 91 % of documented phyllotactic patterns in 650 species of angiosperms and gymnosperms (Jean, 1992), although this type of phyllotaxis is also found in ferns, clubmosses, spike mosses, quillworts and mosses (Okabe, 2024). As a result of the overwhelmingly common nature of Fibonacci spirals, it has been hypothesized that this is the ancestral phyllotaxis for land plants (Jean, 1988). However, it was recently revealed that the phyllotaxis of the Early Devonian lycopod *Asteroxylon mackiei* includes both whorls and non-Fibonacci spirals, suggesting that Fibonacci spiral phyllotaxis is a derived state in leafy plants (Turner *et al.*, 2023), but whether this is true for all leafy plants or only for lycopods remains unclear. Regardless, by the Late Devonian, the progymnosperm stem *Callixylon zalesskyi* had developed 2,3 or 3,5 Fibonacci spiral phyllotaxis (Beck, 1979; Okabe *et al.*, 2019), and in the Late Permian, the early conifer *Ningxiaites specialis* had spiral phyllotaxis of leaves with an unspecified number of parastichies (Wei *et al.*, 2015).

Conifer seed cones are also noteworthy for their typically Fibonacci spiral phyllotaxis, which extends across conifer families. For example, *Masculostrobus rishra*, a possible cheirolepidiaceous pollen cone from the Jurassic of Iran, has been described with a 3,5 Fibonacci spiral phyllotaxis (Barnard, 1968). Likewise, the pinaceous seed cone *Pityostrobus andraei* from the Cretaceous of Belgium shows both 3,5 and 5,8 Fibonacci phyllotaxis (Alvin, 1953). However, Harris noted a regrettable lack of phyllotaxis studies in fossil conifers, particularly in cones (Harris, 1976). This is partly because phyllotaxis can usually be determined only from well-preserved, non-compressed specimens, a problem noted as early as 1873 (Dickson, 1873). Now, with the application of newer methods, such as microcomputed X-ray or synchrotron tomography (micro-CT), phyllotaxis can be determined from the internal seed arrangement in three-dimensionally preserved cones (e.g. Gee, 2013; Gee *et al.*, 2014; Steart *et al.*, 2014), which might be particularly useful if cones exhibit a high degree of surficial abrasion on their outer surfaces. Nevertheless, even with these new imaging methods, in the most detailed morphological and anatomical studies, fossil cones are often described only as having helical or spiral phyllotaxis, without specifying the number of spirals, their chirality or the rotation of the spirals. For many years, this has been true for the well-preserved seed cones of *Araucaria mirabilis*, which show a detailed internal structure with X-ray tomography that is as exquisite as the preservation of their outer appearance.

*Araucaria mirabilis* seed cones were initially discovered by Windhausen in 1919 (Windhausen, 1931) in what is now called the Bosques Petrificados de Jaramillo National Park, formerly known as the Cerro Cuadrado Petrified Forest, in Patagonia, Argentina. The seed cones were first described by Spegazzini (1924) as *Araucarites mirabilis*, but taxonomic revisions of *Araucarites mirabilis* and *Proaraucaria mirabilis* (Wieland, 1935) reassigned the species to *Araucaria mirabilis* (Calder, 1953). The cellular-level preservation (Stockey, 1975) of these 165- to 161-Myr-old (Spalletti *et al.*, 1982) cones has allowed for the study of their gross morphology and internal anatomy. In particular, the tissues of seeds and embryos have been well described (Stockey, 1975), as has the cellular structure of the axis and the vascular system (Stockey, 1977). Externally, the fossilization of the seed cones is excellent and allows for the study of the bract/ovuliferous scale complexes to understand the details of their spiral phyllotaxis and, to some degree, the internal construction of the cones.

Wieland’s (1935) description of *Proaraucaria mirabilis* does not refer to the arrangement of the seeds or bract/ovuliferous scale complexes. Calder’s (1953) description mentions only that the scales are organized in a ‘close spiral’ with ∼55 orthostichies (vertically arranged rows). In her description of *A. mirabilis* seeds and embryos, Stockey (1975) noted numerous cone scales arranged spirally around the central axis of each cone.

A deeper analysis of the phyllotaxis of *A. mirabilis* cones documented between 8 and 21 parastichies in six specimens (Gee, 2013). Follow-up work in an unpublished thesis by Weiser (2015) found similar numbers, ranging from 10 to 21 parastichies, with 18–42 seeds in each. In both studies, only the clockwise parastichies of each cone were counted. Noll and Kunzmann (2020) took a closer look at the phyllotaxis of *A. mirabilis* that included clockwise, anticlockwise and pseudoparastichies, within a taxonomic context. There, the description of *A. mirabilis* cones was amended to encompass 16–22 clockwise parastichies, 11–13 anticlockwise parastichies and 15–34 pseudoparastichies (Noll and Kunzmann, 2020). Additionally, Noll and Kunzmann (2020) established or recombined three *Araucaria* additional species from the Bosques Petrificados de Jaramillo National Park: *Araucaria stockeyana*, *Araucaria minima* and *Araucaria cuneoi*. Although these distinctions were made primarily based on the curvature of the distal tips of the bract/scale complexes and the number of bract/scale complexes per cone, they also included the phyllotaxis of each seed cone species, with *A. cuneoi* possessing 18–34 parastichies, *A. stockeyana* having 8 clockwise and 13 anticlockwise parastichies, and *A. minima* likewise consisting of 8 clockwise and 13 anticlockwise parastichies.

Despite this work, documenting the phyllotaxis does not address the biological function, if any, of these patterns. It has been hypothesized that spiral phyllotaxis of foliar elements on a shoot minimizes the overlap between them, allowing maximum light exposure to each leaf (Niklas, 1988; Pearcy and Yang, 1998; King *et al.*, 2004), although other studies have contradicted this finding (Valladares and Brites, 2004; Sarlikioti *et al.*, 2011; Strauss *et al.*, 2020). Regardless, although immature conifer seed cones photosynthesize (Koppel *et al.*, 1987; Dick *et al.*, 1990; Wang *et al.*, 2006), this is not the primary function of a seed cone, and therefore optimizing light capture is unlikely to be the major advantage of conifer seed cone structure. Although phyllotaxis in seed cones might be a vestigial trait resulting from homology of the seed cone to other foliar elements, cone phyllotaxis might also have implications for effective seed packing and cone seediness, which is the number of seeds that can be packed in a cone. It has been recognized in recent cones that efficient or optimal seed packing has an effect on seed mass, cone size and even germination and predation (DeSoto *et al.*, 2017).

Here, we analyse the phyllotaxis of the 165-Myr-old seed cones of *A. mirabilis* using both non-destructive micro-CT and gross morphology to determine whether phyllotaxis or other morphometric characters, such as axis or cone proportions, had an influence on optimal seed cone packing and cone seediness on araucarian seed cones in the Jurassic. Of these, axis length, cone width, ontogenetic chirality and degree of parastichy rotation all influence seediness.

### Previous studies on phyllotaxis

Phyllotaxis has been studied rigorously by mathematicians and botanists alike. Scores of publications have been produced analysing the mathematical properties and theorizing about the biological catalyst for the patterns that are observed (e.g. Kilmer, 1971; Mitchison, 1977; Douady and Couder, 1992; Bernasconi and Boissonade, 1997; Koch *et al.*, 1998; Lee and Levitov, 1998; Kunz, 2001; Meinhardt, 2003; Yamada *et al.*, 2004; Korn, 2008; Mughal and Weaire, 2017; Okabe *et al.*, 2019). The most common form of phyllotaxis among all clades of plants is spiral, also known as helical (Jean, 1992; Okabe *et al.*, 2019), which is evident in the patterns of sunflower flowering heads, succulent stems and conifer seed cones (Rutishauser and Peisl, 2001).

**Fig. 1.**
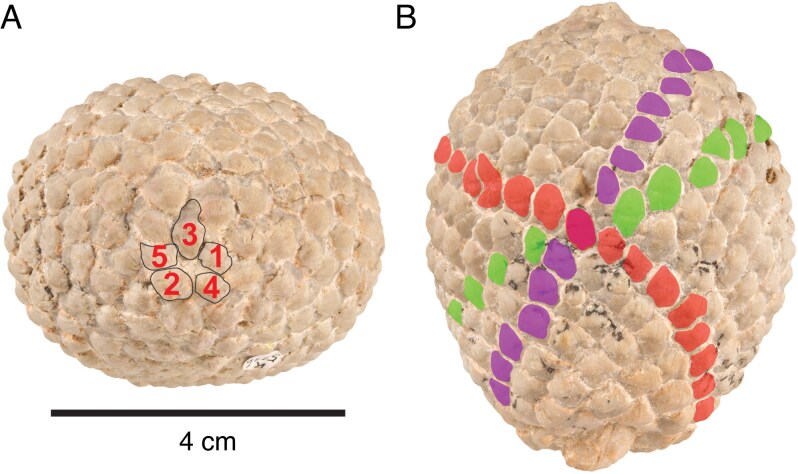
Parastichy and ontogenetic spirals. (A) The ontogenetic spiral represented by the five youngest ovuliferous scales at the apex of K5636 (outlined and numbered), with 1 being the youngest, 2 the second youngest, and so on, indicating a clockwise direction of development. (B) Clockwise parastichy (green), anticlockwise parastichy (red) and pseudoparastichy (purple) of K5636. Photographs by Manuel Kunz. Scale bar: 4 cm.

In spiral phyllotaxis, each bract/scale complex in a seed cone grows sequentially at the apical meristem, separated from the previous bract/scale by a divergence angle of ∼137.5°. This causes all bract/scales to grow in a spiral without making contact with the preceding or subsequent one (Fig. 1A). This spiral is typically referred to as the genetic spiral (Jean, 1994; Korn, 2008). However, we prefer to call this the ontogenetic spiral because it directly reflects the developmental growth, or ontogeny, of the seed cone. The ontogenetic spiral in a cone can be left- or right-handed, and the handedness, or chirality, can be determined by finding the youngest scale at the apical meristem, then finding the second-youngest scale. The second-youngest scale will be 137.5° from the youngest scale, either to the left or to the right, indicating the growth direction of the ontogenetic spiral. The biological explanation for this growth pattern remains debated, but several mechanisms might be involved, because meristematic structure differs across plant clades (Gola and Banasiak, 2016).

As the ontogenetic spiral develops, visual spirals known as contact parastichies are formed by the overlap of leaves or bract/scale complexes in both clockwise and anticlockwise directions (Fig. 1B). These parastichies most often correspond to adjacent numbers in the Fibonacci sequence (1, 1, 2, 3, 5, 8…), e.g. three clockwise and five anticlockwise parastichies (Kilmer, 1971; Mitchison, 1977; Korn, 2008). This is a result of what is well known as the golden angle (137.5°), which separates each new leaf or seed from the one appearing before it (Douady and Couder, 1992; Okabe *et al.*, 2019). However, in some cases, non-Fibonacci spiral numbers may appear, such as ‘duplicate’ Fibonacci numbers known as bijugate spirals (2, 4, 6, 10, 16…). Bijugate spirals are uncommon, being found in only 2.5 % of plants, as a result of two ontogenetic spirals forming simultaneously (Korn, 2008). In these, the divergence angle of leaves is 68.8° (Jean, 1994). The Lucas sequence (1, 3, 4, 7, 11, 18…) is also possible, with a divergence angle of 99.5°, but is even rarer, occurring only 1 % of the time (Korn, 2008).

## MATERIALS AND METHODS

### Materials

**Fig. 2.**
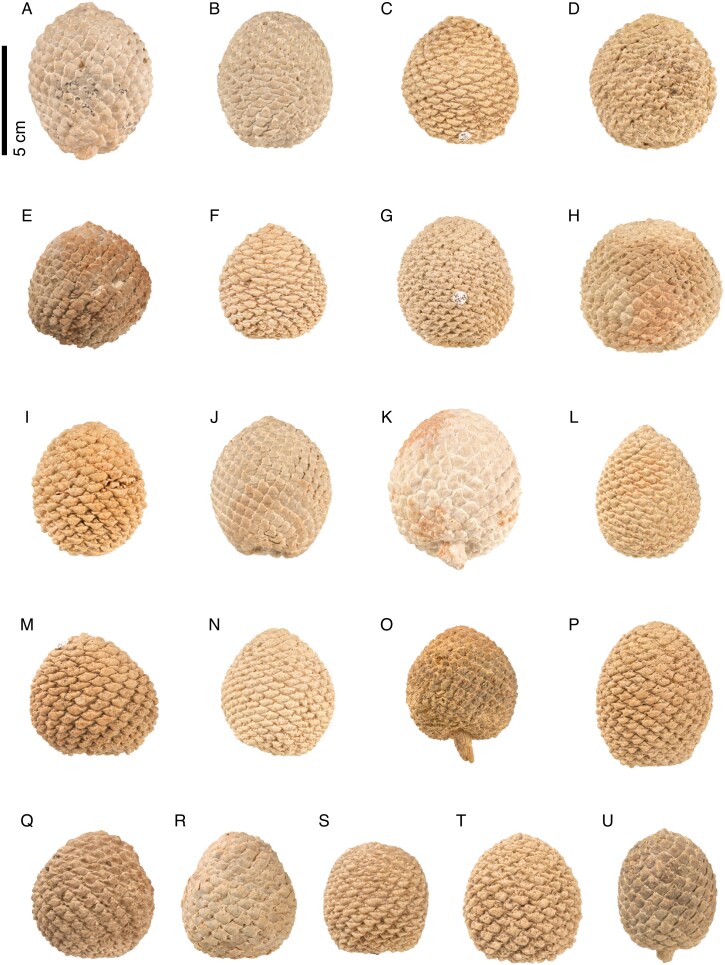
The 21 *Araucaria mirabilis* seed cones from the palaeobotanical collections at the Museum für Naturkunde Chemnitz in Chemnitz, Germany, under investigation here. (A) K5636. (B) K5637. (C) K5638. (D) K5639. (E) K5640. (F) K5641. (G) K5643. (H) K5644. (I) K5645. (J): K5647. (K) K5650. (L) K5651. (M) K5652. (N) K5653. (O) K5677. (P) K5679. (Q) K5680. (R) K5681. (S) K5692. (T) K5695. (U) K5703. Photographs by Manuel Kunz. Scale bar: 5 cm.

Twenty-one silicified *A. mirabilis* seed cones were selected from the palaeobotanical collections at the Museum für Naturkunde Chemnitz in Chemnitz, Germany (Fig. 2). Cones were selected if they were whole and appeared well preserved on the exterior. Of these, the largest available cones were chosen to ensure cone maturity, and several others were selected to expand the range of sizes and shapes in the entire dataset.

*Araucaria mirabilis* seed cones originate from the Bosques Petrificados de Jaramillo National Park, formerly referred to as the Cerro Cuadrado Petrified Forest, in Patagonia, Argentina. Although stratigraphic and coordinate information were not recorded upon collection, *A. mirabilis* seed cones are known to originate almost exclusively from pyroclastic layers of the La Matilde Formation of the Bahía Laura Group (Falaschi *et al.*, 2011), which is Middle Jurassic in age (Bathonian–Callovian; Spalletti *et al.*, 1982). Falaschi *et al.* (2011) present a lithostratigraphic section of the fossiliferous horizons, including tuffs containing seed cones. The depositional environment and geology were described extensively by de Barrio *et al.* (1999).

### Terminology

The terminology for seed cone morphology used here follows Escapa *et al.* (2012) and Escapa and Catalano (2013). Terminology for phyllotaxis is defined in the Supplementary Data Appendix S1.

### Gross morphology

For each cone, the number of clockwise and anticlockwise parastichies, and of the pseudoparastichies (following Noll and Kunzmann, 2020), was determined from its gross morphology (Fig. 1B). Furthermore, the total number of visible bract/scale complexes was counted as a proxy for the approximate number of seeds. The length and width of each seed cone were measured using digital callipers (Gvolatee Digital Vernier Caliper, China). From these data, the length-to-width ratio was calculated to characterize seed cone proportions. Circumference was measured at the widest point of each cone with a soft tape measure. The direction of developmental growth, i.e. the ontogenetic spiral, of the bract/scales was determined through observation, starting from the youngest scales at the cone apex (Fig. 1A).

### Microcomputed X-ray tomography

All seed cones were scanned with a GE phoenix v|tome|xs 180/240 micro-CT (General Electric Measurement and Control Solutions, Wunstorf, Germany) at the Bonn Institute of Organismic Biology, Division of Paleontology, University of Bonn, Germany. The cones were positioned upright, with the proximal end, or cone base, downwards, and scanned using the 240 kV tube. Scan parameters (voltage, current, exposure time, voxel size and number of projections) were optimized individually for each cone to ensure high-quality scans. Further details on the micro-CT scanning process for seed cones were described by Gee (2013).

**Fig. 3.**
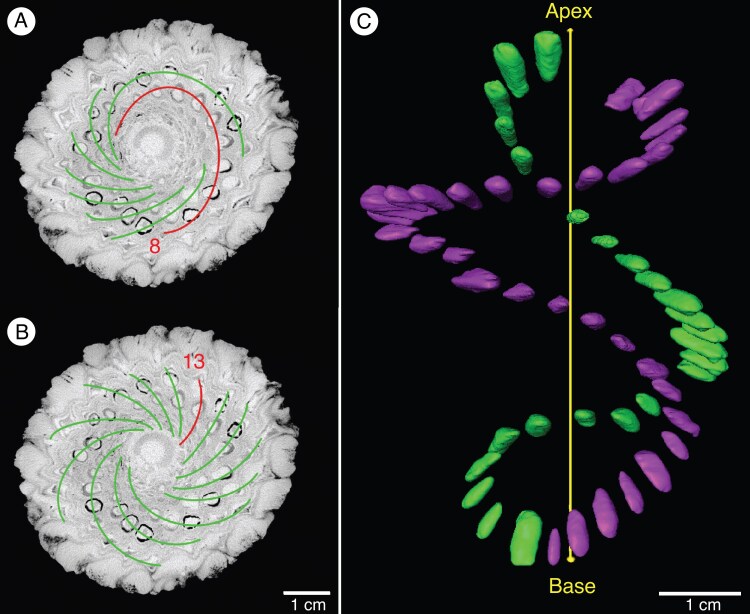
Micro-CT (A, B) and three-dimensional segmented (C) images of K5637 using the seeds as proxies for the bract/scale complexes. (A) Median transverse orthoslice showing eight clockwise parastichies. (B) Same median transverse orthoslice as in A, showing 13 anticlockwise parastichies. (C) Clockwise (green) and anticlockwise (purple) parastichies visualized in three dimensions. Scale bars: 1 cm.

Serial orthoslices (image stacks) were then made using phoenix datos|x (General Electric Measurement and Control Solutions) and VGStudio MAX v.3.2 (Volume Graphics GmbH, Heidelberg, Germany). Using orthoslices in transverse section, the number of clockwise and anticlockwise parastichies in each cone was counted manually (Fig. 3A, B; Adobe Photoshop v.25.5.1) and compared with the number obtained from the examination of the gross morphology. From one image stack, three-dimensional digital reconstructions of seed rows were created with the software Avizo v.8.1.1 (Fig. 3C) (FEI SAS, ThermoFisher Scientific, Waltham, MA, USA, and Konrad-Zuse-Zentrum für Informationstechnik, Berlin, Germany). With Avizo, the maximum length and width of the central axis of each cone were measured. For the segmentation process, the first seed to appear at the base of the cone was identified (Gee, 2013). From this, one clockwise (as viewed from the cone apex) and one anticlockwise spiral were segmented completely, from base to apex. The total rotation of each parastichy around the axis, henceforth called the degree of parastichy rotation, was then estimated in degrees.

### Statistical analyses

The significance of measured parameters on total seed count and phyllotaxis were calculated with linear regressions and multiple regressions using R Studio v.4.4.2 (R Foundation for Statistical Computing, 2021) with a significance threshold of 0.05. Pearson correlations (*r*) were calculated to determine the strength and direction of interactions. Full statistical results are given in Supplementary Data Tables S1 (linear regressions), S2 (Pearson correlations) and S3 (multiple regressions).

### Data archiving

The raw micro-CT scans have been archived on Morphosource under ID 000774701 (Howell, 2025).

## RESULTS

### Gross morphology

**Fig. 4.**
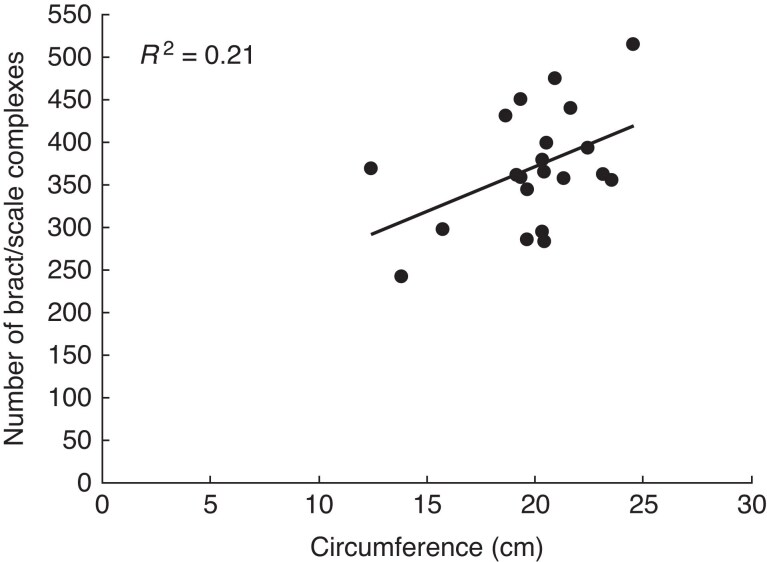
Scatterplot showing the relationship between cone circumference (in centimetres) and the number of bract/scale complexes in *Araucaria mirabilis* seed cones.

The cones studied here range in length from 4.9 to 9.2 cm and in width from 4.0 to 8.3 cm (Table 1). The length-to-width ratios range from 0.8 to 1.3. Only three cones have a length-to-width ratio of less than one, indicating that they are wider than they are high, whereas 18 have lengths greater than their width and are, therefore, taller than they are wide. Cone circumference ranges from 12.0 to 24.5 cm. The number of bract/scale complexes in each cone ranges from 243 to 516. Of the gross morphological features that can be measured, circumference has the greatest influence on the number of bracts/scales (*P* < 0.05; *r* = 0.5; Fig. 4).

**Table 1.** External measurements. The length, width and circumference of each cone in centimetres, the number of bracts/scales on each cone and the length-to-width ratio of each cone are indicators of general seed cone proportions. Values greater than one indicate that the cone is longer than it is wide; values less than one indicate that the seed cone is wider than it is long.

Specimen number	Length (cm)	Width (cm)	Length:width	Circumference (cm)	Number of bract/scales
K5636	9.2	7.2	1.3	21.3	358
K5637	8.2	6.8	1.2	20.5	400
K5638	7.6	7.0	1.1	20.4	366
K5639	7.9	7.5	1.1	22.4	394
K5640	6.5	7.5	0.8	23.5	356
K5641	6.7	6.4	1.0	19.1	362
K5643	8.1	6.8	1.2	20.9	476
K5644	7.5	8.3	0.9	24.5	516
K5645	7.9	6.8	1.2	20.4	284
K5647	8.3	7.0	1.2	21.6	441
K5650	8.8	7.6	1.2	23.1	363
K5651	7.9	6.4	1.2	19.6	345
K5652	6.4	6.8	0.9	20.0	295
K5653	7.7	6.7	1.1	20.3	380
K5677	6.1	5.8	1.1	19.3	451
K5679	7.6	6.1	1.2	18.9	432
K5680	6.5	6.4	1.0	19.3	359
K5681	6.8	6.3	1.1	19.6	286
K5692	4.9	4.0	1.2	12.0	370
K5695	6.1	5.9	1.0	16.3	298
K5703	5.3	4.4	1.2	13.8	243

**Fig. 5.**
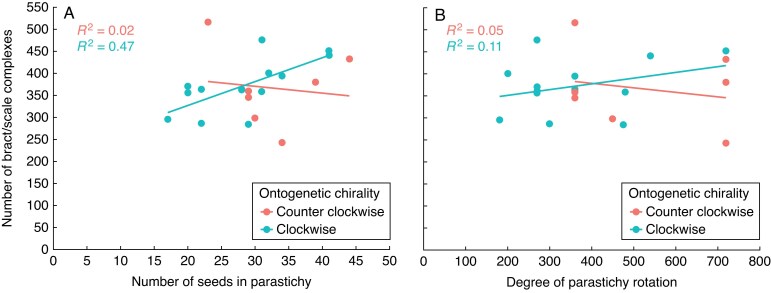
Scatterplots showing the influence of ontogenetic chirality on the number of bract/scale complexes in *Araucaria mirabilis* seed cones as a result of the number of seeds in a parastichy (A) and the degree of parastichy rotation (B).

Fourteen cones have clockwise ontogenetic spirals, and the other seven have anticlockwise ontogenetic spirals (Table 2). Although the direction of the ontogenetic spirals is not significantly correlated with the total number of bract/scale complexes per cone (*P* > 0.5, *r* = 0.2), which is directly related to cone seediness (DeSoto *et al*., 2017), it does have an interaction with other characteristics. The number of seeds in a parastichy does not significantly influence cone seediness (*P* > 0.1, *r* = 0.3); however, this correlation becomes statistically significant when associated with clockwise ontogenetic chirality (multiple regression: *P* < 0.05, *r* = 0.3; Fig. 5A). Likewise, the degree of parastichy rotation is not significant (*P* > 0.5) until also associated with clockwise ontogenetic chirality (multiple regression: *P* < 0.05, *r* = 0.2; Fig. 5B). Furthermore, a larger circumference has no significant correlation with an increased number of clockwise parastichies (*P* = 0.3, *r* = 0.07), but when the cone has clockwise ontogenetic chirality, this correlation becomes significant, although weak (multiple regression: *P* < 0.05, *r* = 0.07).

**Table 2.** External features. The number of parastichies visible on the cone surface. Ontogenetic direction is the clockwise (CW) or anticlockwise (ACW) ontogenetic chirality, based on the developmental order of the youngest bract/scales at the cone apex.

Specimen number	Clockwise parastichies	Anticlockwise parastichies	Pseudoparastichies	Ontogenetic chirality
K5636	11	16	26	CW
K5637	13	21	37	CW
K5638	13	21	37	ACW
K5639	13	21	9	CW
K5640	13	21	34	CW
K5641	13	21	8	CW
K5643	16	10	26	CW
K5644	21	13	30	ACW
K5645	10	14	21	CW
K5647	11	18	29	CW
K5650	21	13	35	CW
K5651	13	21	9	ACW
K5652	8	13	21	CW
K5653	10	16	26	ACW
K5677	13	21	34	CW
K5679	11	16	26	ACW
K5680	13	21	34	ACW
K5681	13	21	7	CW
K5692	13	21	34	CW
K5695	13	21	34	ACW
K5703	13	21	27	CW

The number of clockwise parastichies in all cones ranges between 8 and 21, while anticlockwise parastichies fall between 10 and 21 (Table 2). The most common parastichy pair is 13,21, present in 14 of 21 seed cones (64 %). A greater number of clockwise parastichies has a weak correlation with greater cone seediness (*P* < 0.05, *r* = 0.1), but there is no correlation at all with the number of anticlockwise parastichies (*P* = 0.92, *r* = −0.02). Furthermore, pseudoparastichies, which range between 7 and 37 (Table 2), are not significantly correlated with any other morphological features. The numbers of clockwise parastichies and anticlockwise parastichies have an inverse correlation (*P* < 0.05, *r* = −0.5).

### Internal characteristics

**Fig. 6.**
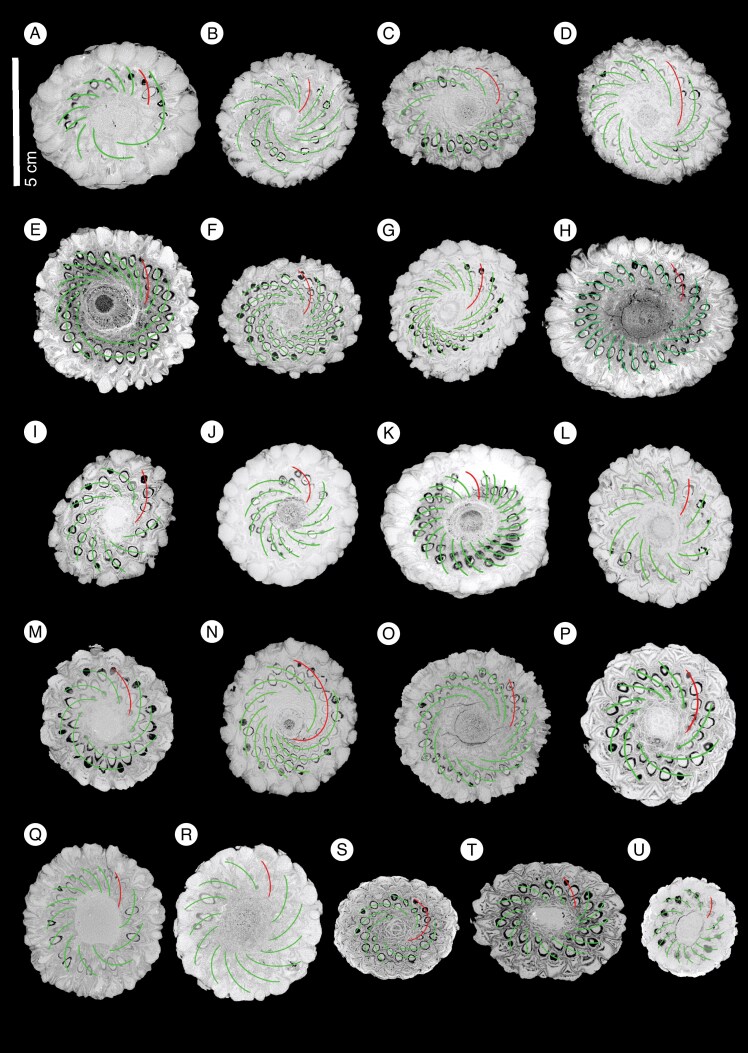
Clockwise parastichies on transverse micro-CT orthoslices. (A) K5636 = 10. (B) K5637 = 13. (C) K5638 = 13. (D) K5639 = 13. (E) K5640 = 13. (F) K5641 = 13. (G) K5643 = 16. (H) K5644 = 21. (I) K5645 = 10. (J) K5647 = 11. (K) K5650 = 21. (L) K5651 = 13. (M) K5652 = 8. (N) K5653 = 10. (O) K5677 = 13. (P) K5679 = 10. (Q) K5680 = 13. (R) K5681 = 13. (S) K5692 = 8. (T) K5695 = 13. (U) K5703 = 13. Scale bar: 5 cm.

**Fig. 7.**
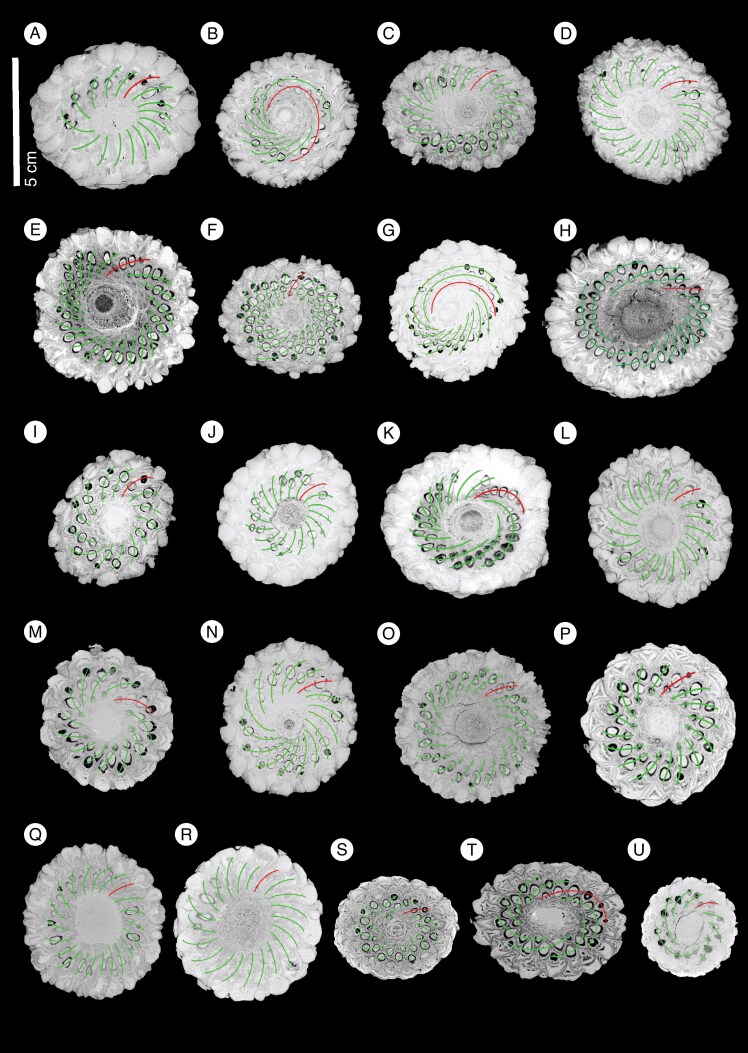
Anticlockwise parastichies on transverse micro-CT orthoslices. (A) K5636 = 16. (B) K5637 = 8. (C) K5638 = 21. (D) K5639 = 21. (E) K5640 = 21. (F) K5641 = 21. (G) K5643 = 10. (H) K5644 = 13. (I) K5645 = 14. (J) K5647 = 18. (K) K5650 = 13. (L) K5651 = 21. (M) K5652 = 13. (N) K5653 = 16. (O) K5677 = 21. (P) K5679 = 16. (Q) K5680 = 21. (R) K5681 = 21. (S) K5692 = 13. (T) K5695 = 8. (U) K5703 = 8. Scale bar: 5 cm.

In most cones, the number of parastichies recognized through micro-CT is the same as the number of parastichies observed externally (Figs 6 and 7; Table 3); however, for six cones (K5636, K5637, K5679, K5692, K5695 and K5703), there are differences between the two counts (Tables 2 and 3). For clockwise parastichies, the degree of rotation ranges from 200° to 720° between cones, with the most common rotation being 360° (*n* = 8), as shown in Fig. 3C. The anticlockwise parastichies rotate between 260° and 900°, with the most common being 360° (*n* = 12). The number of parastichies has a moderate correlation with the degree of parastichy rotation (*P* = 0.05, *r* = 0.4). The correlation with the number of seeds in a spiral is even more significant (*P* < 0.001, *r* = 0.6).

**Table 3.** Internal features observed from micro-CT scanning and Avizo segmentation.

Specimen number	Clockwise parastichies			Anticlockwise parastichies		
	Number of parastichies	Rotation (°)	Number of seeds	Number of parastichies	Rotation (°)	Number of seeds
K5636	10	360	23	16	360	27
K5637	13	200	32	8	540	22
K5638	13	360	28	21	900	48
K5639	13	360	34	21	260	20
K5640	13	270	20	21	360	34
K5641	13	360	28	21	360	33
K5643	16	270	31	10	400	28
K5644	21	360	23	13	360	38
K5645	10	475	29	14	360	24
K5647	11	540	41	18	320	28
K5650	21	360	22	13	360	38
K5651	13	360	29	21	720	37
K5652	8	180	17	13	360	25
K5653	10	720	39	16	300	25
K5677	13	720	41	21	360	26
K5679	10	720	44	16	360	28
K5680	13	360	29	21	360	26
K5681	13	300	22	21	720	34
K5692	8	270	20	13	360	32
K5695	13	450	30	8	360	25
K5703	13	720	34	8	300	19

**Fig. 8.**
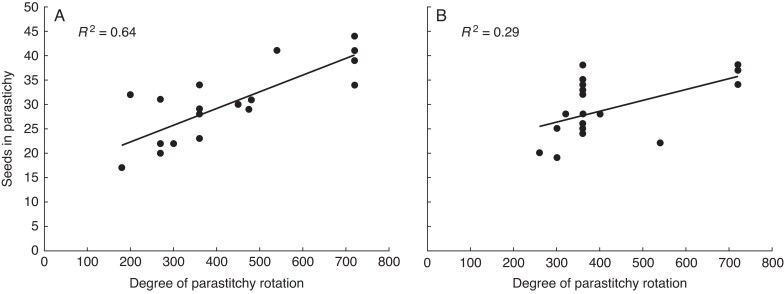
Scatterplots showing the relationship between the degree of parastichy rotation and the number of seeds in parastichies in *Araucaria mirabilis* seed cones. (A) Clockwise parastichies. (B) Anticlockwise parastichies.

**Fig. 9.**
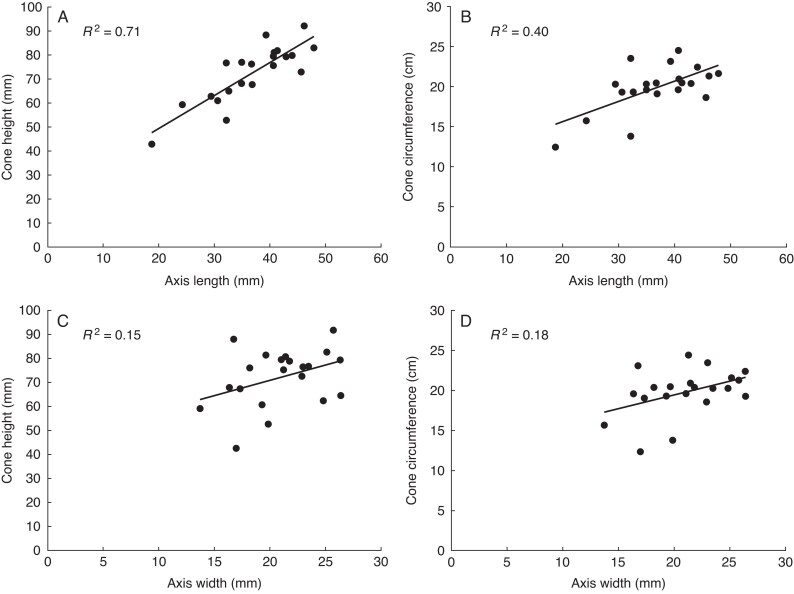
Scatterplots showing the relationships between axis proportions and seed cone proportions in *Araucaria mirabilis* seed cones. (A) Axis length and cone length. (B) Axis length and cone circumference. (C) Axis width and cone length. (D) Axis width and cone circumference.

The number of seeds in any given spiral ranges from 17 to 48 (Table 3). This number is strongly correlated with the degree of the spiral, with more seeds occurring in parastichies with a greater degree of rotation (clockwise: *P* < 0.001, *r* = 0.8, Fig. 8A; anticlockwise: *P* < 0.001, *r* = 0.7, Fig. 8B.). The length of the central axes of the seed cones ranges between 13.8 and 26.4 mm, and the width is between 18.8 and 47.8 mm (Table 4). In all cone specimens, the axis is wider than it is long. A longer axis length is correlated with a longer cone (*P* < 0.005, *r* = 0.7, Fig. 9A), in addition to broader circumference (*P* < 0.01, *r* = 0.6, Fig. 9B) and cone width (*P* < 0.01, *r* = 0.7), whereas axis width is not significantly correlated with any of these (cone height: *P* > 0.05, *r* = 0.3, Fig. 9C; cone circumference: *P* > 0.05, *r* = 0.5, Fig. 9D). However, unlike axis length, axis width is weakly correlated with the degree of parastichy rotation, with a wider axis allowing for longer rotations (*P* = 0.05, *r* = 0.1).

**Table 4.** Central cone axes parameters. The length and width of the central axis of each cone and the length-to-width ratio of each cone are indicators of general seed cone shape. Values greater than one indicate that the axis is longer than it is wide; values less than one indicate that the axis is wider than it is long.

Specimen number	Length (mm)	Width (mm)	Length:width
K5636	25.7	46.1	0.6
K5637	19.6	41.3	0.5
K5638	18.2	36.7	0.5
K5639	26.3	44.0	0.6
K5640	23.0	32.1	0.7
K5641	17.3	36.8	0.5
K5643	21.4	40.7	0.5
K5644	21.3	40.6	0.5
K5645	21.8	42.9	0.5
K5647	25.1	47.8	0.5
K5650	16.7	39.3	0.4
K5651	21.0	40.6	0.5
K5652	24.8	29.4	0.8
K5653	23.4	34.9	0.7
K5677	19.3	30.6	0.6
K5679	22.9	45.6	0.5
K5680	26.4	32.6	0.8
K5681	16.4	34.9	0.5
K5692	17.0	18.8	0.9
K5695	13.8	24.2	0.6
K5703	19.9	32.6	0.6

## DISCUSSION

Based on the species characteristics outlined by Noll and Kunzmann (2020), all cones in this study pertain to *A. mirabilis*. Firstly, micro-CT orthoslices indicate that the distal tips of the bract/scale complexes in all cones point upwards, a major diagnostic feature defined by Noll and Kunzmann (2020) for *A. mirabilis*. Secondly, all cones have between 200 and 700 cone scales (bract/scale complexes; Table 1), which excludes the species *A. stockeyana* (>700 bract/scale complexes), *A. cuneoi* (>700 bract/scale complexes) and *A. minima* (<200 bract/scale complexes).

### Cone and axis shape

Among the morphometrics analysed here, it is notable that the length of the cone axis is related to the width, but not the length, of the seed cones themselves. Conversely, the width of the cone axis is associated with the length, but not the width, of the seed cones. Given that circumference is directly associated with greater seediness, one possible strategy for increasing the number of seeds in a cone would be to increase axis length. However, a wider axis will also increase this number. Thus, an axis that is both tall and wide concretely allows for more bract/scale complexes (Fig. 6). Because of this, the distinctive spherical to ellipsoid cone shape for *A. mirabilis* is ideal for maximizing seediness and, therefore, for reproductive potential.

In general, Araucariaceae seed cones tend towards sphericity (Stockey, 1982), unlike Pinaceae, which tend towards elongate forms (Page, 1990). For example, Forde (1964) found that *Pinus radiata* seed cones became proportionally narrower as cones became longer. However, Forde’s (1964) data across 1500 cones also showed that within this species, populations whose cones had lower length-to-width ratios possessed more cone scales. Besides seediness, seed cone evolution is influenced by several functional requirements during their ontogeny: pollen reception, seed protection and seed dispersal, which may have conflicting optimal morphologies (Huntsman and Leslie, 2024). Although both the Pinaceae and the Araucariaceae developed robust, woody cones to protect seeds from predation (Leslie, 2011), the Araucariaceae, including *A. mirabilis*, might have developed an emphasis on overall seediness through sphericity, whereas the Pinaceae might have been more strongly influenced by one of the other functional requirements that benefitted from an elongate morphology.

### Ontogenetic chirality

Ontogenetic spiral chirality might also contribute to cone seediness. Although ontogenetic chirality is not correlated directly with the total number of ovuliferous scales in the seed cones, there is significant covariance with other morphometrics. For instance, when the ontogenetic spiral is clockwise, wider circumferences become associated, albeit weakly, with a greater number of clockwise parastichies (multiple regression: *P* < 0.05, *r* = 0.07). In general, a higher number of clockwise parastichies is weakly correlated with greater seediness (*P* < 0.05, *r* = 0.1). Most significantly, clockwise ontogenetic chirality is associated with tighter seed spirals (*P* < 0.05, *r* = 0.2) that have more seeds (*P* < 0.05, *r* = 0.3). This, in turn, might lead to greater cone seediness.

At this time, it is unclear whether there is a structural character, such as seed shape or symmetry, that would pack more efficiently in a particular direction of growth, or whether collection bias is a factor. Because the precise coordinates of specimen collection are unknown, we cannot rule out that the cones in this study are biased towards clockwise chirality because they are part of a subset that happens to favour this orientation. However, the collecting of *A. mirabilis* cones by different people over many years also makes it more likely that the cones were not procured with a particular collection bias, but non-selectively from different locations and various horizons, then grouped later.

Studies quantifying spiral chirality in angiosperms consistently find that it is randomly developed and equally distributed (for a review, see Galloway, 1989). However, there have been very few studies quantifying cone chirality in gymnosperms. Some have found that individual trees bore cones that spiralled in both directions (Compton, 1912; Forde, 1964), but Galloway (1989) notes that the ratios of clockwise to anticlockwise spirals vary between species. For example, *Pinus austriaca* more commonly shows a clockwise orientation (≤59 %; Compton, 1912), whereas *Pinus laricio* favours anticlockwise orientations (≤79 %; Compton, 1912). However, *Pinus radiata* has very close to 50 % of both orientations across an entire population, while individual trees have cones with chirality in one direction (Forde, 1964). These studies have been limited to the genus *Pinus*, however. Studies in other gymnosperm taxa, particularly of the genus *Araucaria*, would provide more information about whether chirality in this genus favours clockwise orientations, as is observed here.

### Phyllotaxis in *Araucaria mirabilis* cones

Externally, the number of parastichies here ranges from 8 to 21 (Table 2), which concurs with Noll and Kunzmann (2020), who found that *A. mirabilis* cones have 8–22 parastichies, and Gee (2013), who documented 8–21 parastichies. The cones studied here have 7–37 pseudoparastichies (Table 2), whereas those examined by Noll and Kunzmann (2020) had 25–34. Our study encompasses a larger sample size (*n* = 21) than the study by Noll and Kunzmann (2020; *n* = 4), which might account for the wider range of pseudoparastichies. In the Araucariaceae, the fossil species *A. minima* and *A. stockeyana* are described as having 5,8 phyllotaxis by Noll and Kunzmann (2020). In seed cones of living species of *Araucaria*, Weiser (2015) counted parastichies in one direction, not both, on a limited number of cones, but found *Araucaria laubenfelsii* (*n* = 2), *A. rulei* (*n* = 2), *A. muelleri* (*n* = 3) and *A. heterophylla* (*n* = 1) all to have 13 parastichies. *Araucaria cunninghamii* (*n* = 2) has eight parastichies and *A. araucana* has 21 (*n* = 1; Weiser, 2015). Despite the low number of specimens for each species in these three studies, the seed cones of the genus *Araucaria* appear to show a broad variation in the number of their parastichies, but they tend towards higher Fibonacci pairs, with a minimum of 5,8 phyllotaxis. This contrasts with the members of family Pinaceae, in which multiple genera and species have only three spirals of seeds in fossil and living species (Gee, 2013; Gee *et al.*, 2014).

It is well documented that plants exhibiting spiral phyllotaxis, including seed cones, have parastichy pairs that follow the Fibonacci sequence (1, 1, 2, 3, 5, 8, 12, 21…) (Mitchison, 1977; Douady and Couder, 1992; Okabe *et al.*, 2019). It is typical to have parastichy pairs such as 5,8 or 13,21, as seen in the cones sampled here (Table 2). All but six seed cones observed here follow this pattern. The rare variant of the Fibonacci sequence called the Lucas sequence (Korn, 2008), which was mentioned earlier, is shown by cone K5647 in its phyllotaxis of 11,18. Four cones (K5636, K5643, K5653 and K5679) have 10,16 phyllotaxis internally. These four cones instead follow a duplicated Fibonacci series, also known as bijugate spirals, that result from the cone possessing two opposite ontogenetic spirals (Korn, 2008). This might be an aberrant growth pattern, but in some conifer species, such as *Cephalotaxus*, bijugacy is the primary phyllotactic pattern for branching and leaf growth (Tomlinson and Zacharias, 2001). Noll and Kunzmann (2020) also observed one *A. mirabilis* cone with 11,16 phyllotaxis. This cone might also have exhibited bijugacy, although it shows 11,16 parastichies instead of 10,16. Two cones studied here show 11,16 externally but 10,16 internally. This apparent discrepancy might result from an aberration noted by Fierz (2015), in which a parastichy sometimes appears or disappears partway through the cone, and might indicate a transition between patterns during ontogeny.

Fierz (2015) found that 97 % of *Pinus nigra* and 78 % of *Larix decidua* cones followed the Fibonacci sequence. They hypothesized that deviations such as bijugacy or the Lucas sequence result from the size of the primordia in the early stages of seed cone development. Specifically, non-Fibonacci patterns might result from an atypical ratio between primordium size and meristem size, the ontogeny of which might be a result of genetics (Zagórska-Marek and Szpak, 2008) or the environment (Vakarelov, 1998). This might explain K5645, in which the phyllotaxis (10,14) does not match any documented numerical patterns (cf. Jean, 1994). Furthermore, the number of parastichies can change throughout ontogeny (e.g. *Picea abies*; Rutishauser, 1998), but the chirality of the ontogenetic spiral itself does not change (Meicenheimer, 1998). Therefore, although parastichy numbers can characterize certain taxa (Gee, 2013), the variation in parastichy numbers observed in *A. mirabilis* does not necessarily suggest that multiple taxa can be distinguished based on phyllotaxis alone. Only a range of parastichy pairs, ranging from 8,13 to a maximum of 13,21, can be established for *A. mirabilis* seed cones (Table 2; Gee, 2013; Noll and Kunzmann, 2020).

Internally, it was observed that the angles of rotation of the parastichies range from 200°, less than a full rotation of 360°, to 900°, which is approximately two-and-a-half rotations around the axis. Both clockwise and anticlockwise parastichies were highly variable in the degree of their rotations, but the most common was one full rotation of 360°, occurring in 8 clockwise and 12 anticlockwise parastichies. Araucariaceae seed cones have a wide range of spiral rotations. In recent seed cones, Gee (2013) noted a rotation in *A. araucana* as low as 0°, producing vertical parastichies between the base and apex with no turn around the axis, known as orthostichies (cf. Okabe, 2024), in addition to a rotation in a fossil *Araucaria* seed cone from Wyoming as high as 360° (Gee, 2013). Burlingame (1914) recorded what he called *Araucaria brasiliensis* (current synonymy: *A. angustifolia*), with spirals exceeding 540° of rotation. No other studies have quantified the degree of parastichy rotation within the Araucariaceae, making the 900° spiral observed here the greatest known in the family. However, as documented by Gee (2013), some Pinaceae seed cones exhibit spirals with ≤1440° of rotation. Further studies in the seed spiral rotations of the Araucariaceae would shed light on whether the high variation seen in *A. mirabilis* is unusual and on how the degree of rotation affects optimal cone packing.

### Seediness and seed packing

Seed mass might also alter the packing of seeds in a cone (DeSoto *et al.*, 2017), but this was not considered here owing to the fossil nature of the specimens. In general, however, greater cone seediness is correlated with overall smaller seeds, which has biological trade-offs, because larger seeds typically germinate and establish more successfully (Moles and Westoby, 2004); however, it should be noted that smaller seeds are less likely to be eaten (Gómez, 2004). Greater seediness also corresponds to greater cone or fruit size (Valido *et al.*, 2011), which is observed here as the positive correlation between seediness and circumference, and between axis length and seediness. Seed cone packing might also be influenced by other required functions of conifer seed cones, including pollination efficiency, seed protection and dispersal efficiency (Leslie, 2011; Losada *et al.*, 2019). For example, within Pinaceae, cones that disperse via disintegration or abscission have larger seeds on average than those that disperse via ‘flexing’ that rely on wind to carry their seeds (Losada *et al.*, 2019). Stockey (1975) suggested that the Middle Jurassic *A. mirabilis* seeds might have shed from the bract/scale complex, as in living *Araucaria bidwillii*. The shedding of seeds from bract/scale complexes was also noted by Gee and Tidwell (2010) for the Late Jurassic cones of *Araucaria delevoryasii*. Gee and Tidwell (2010) also noted the occurrence of an abundance of detached bract/scale complexes, suggesting that the seed cones of *A. delevoryasii* used the same method of seed dispersal as the ‘shatter cones’ of *A. bidwillii* do today, although *A. bidwillii* seeds additionally shed from the ovuliferous scale (Stockey, 1982).

**Fig. 10.**
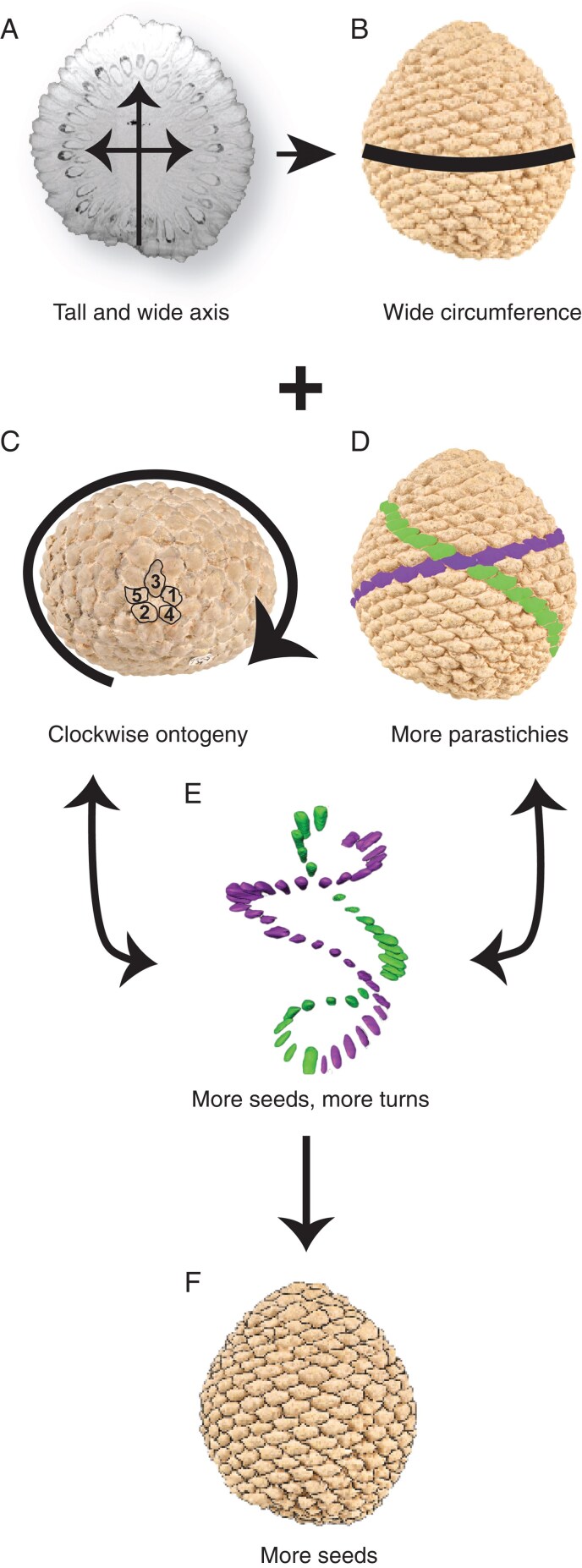
Hypothetical schematic figure visualizing the potential influence of different characteristics on seed packing. An axis that is both taller and wider (A) leads to a wider circumference (B). In combination with a clockwise ontogeny (C) and more parastichies in the cone (D), the number of seeds in each spiral and their degree of rotation around the axis (E) may increase. Altogether, these characteristics may increase the number of seeds in each cone (F).

In summary, given the information available from the morphometrics and seed arrangement of *A. mirabilis* cones, the characteristics that are most significantly correlated with cone seediness and might influence optimal cone packing in *A. mirabilis* are a longer cone axis and a clockwise ontogenetic spiral, with a lesser degree of correlation from a greater number of parastichies. Taken together, *A. mirabilis* seed cones could have used several possible strategies for increasing seediness simultaneously: increase circumference with a wider, longer axis (Fig. 10A, B); grow in a clockwise direction (Fig. 10C); and have a higher number of clockwise parastichies (Fig. 10D). Together, these characteristics might lead to longer spirals with more seeds (Fig. 10E), which, in turn, could increase seediness (Fig. 10F).

### Conclusion

A complex suite of morphological features might explain the phyllotaxis and seediness of *A. mirabilis* seed cones, but only a few of these features stand out as the most significant. The first of these is the length and width of the central axis of the cone. Both an increased axis length and an increased axis width are associated with a higher number of bract/scale complexes in the cones; thus, the spherical to ellipsoid shape characteristic of *A. mirabilis* might be ideal for maximizing seed capacity. Secondly, two-thirds of the *A. mirabilis* seed cones in this study were observed to have a clockwise ontogenetic chirality, which is correlated with tighter spirals with more seeds in them and, therefore, greater seediness in the cones. Based on the correlations observed in this study, clockwise ontogenetic chirality is an advantageous feature of *A. mirabilis* seed cones.

## Supplementary Material

mcaf325_Supplementary_Data

## SUPPLEMENTARY DATA

Supplementary data are available at *Annals of Botany* online and consist of the following. Appendix S1: glossary. Table S1: results for linear regression analyses showing *P-*value, correlation coefficient (*r*), *t-*statistic (*t*), degrees of freedom (d.f.), the lower 95% confidence interval (95% CI [L]), the upper 95% confidence interval (95% CI [U]) and the *F-*statistic. Table S2: Pearson correlation coefficients between measured variables used in the multiple linear regression models. Table S3: summary of multiple linear regression models.
