# Supplementary material for: Phyllotaxis, ontogeny and CT imaging: old and new approaches to understanding optimal seed packing in Middle Jurassic Araucaria mirabilis cones
Source: Ann Bot. 2025 Dec 26;137(6):1817–31. doi: 10.1093/aob/mcaf325 (PMC13275025; doi:10.1093/aob/mcaf325)
Supplement: mcaf325_Supplementary_Data [file mcaf325_supplementary_data.docx]

SUPPLEMENTAL TABLES – STATISTICS

Supplemental Table S1. Results for linear regression analyses showing *p-*value, correlation coefficient (r), *t-*statistic (t), degrees of freedom (df), the lower 95% confidence interval (95% CI [L]), the upper 95% confidence interval (95% CI [U]), and the *F-*statistic. CWSeedNo: number of seeds in the segmented CW parastichy. CCWSeedNo: number of seeds in the segmented CCW parastichy. CWDeg: degree of rotation of the CW parastichy. CCWDeg: degree of rotation of the CCW parastichy. AxisWidth: width of cone axis. AxisLength: length of cone axis. CWNo: number of clockwise parastichies. CCWNo: number of counterclockwise parastichies. PseudoNo: number of pseudoparastichies. TotalSeed: number of bract/scale complexes in cone. Length: cone length. Width: cone width. Circumference: cone circumference. OntoDir: direction of ontogenetic growth.

| Variable x | Variable y | *p-*value | r | t | df | 95% CI [L] | 95% CI [U] | *F-*statistic |
| --- | --- | --- | --- | --- | --- | --- | --- | --- |
| TotalSeed | Length | 0.1636 | 0.315471 | 1.4491 | 19 | -0.1345376 | 0.6576025 | 2.1 |
| TotalSeed | Width | 0.0556 | 0.423722 | 2.0391 | 19 | -0.0097473 | 0.7231364 | 4.158 |
| TotalSeed | Circumference | 0.0384 | 0.454618 | 2.2248 | 19 | 0.02853084 | 0.7409013 | 4.95 |
| TotalSeed | CWNo | 0.7038 | -0.08819 | -0.38592 | 19 | -0.5008113 | 0.3570907 | 0.149 |
| TotalSeed | CCWNo | 0.9199 | -0.02337 | -0.10191 | 19 | -0.4505145 | 0.4124752 | 0.01 |
| TotalSeed | CWDeg | 0.8544 | 0.042626 | 0.18597 | 19 | -0.3963545 | 0.4657423 | 0.035 |
| TotalSeed | CCWDeg | 0.5504 | -0.13816 | -0.60804 | 19 | -0.5377701 | 0.3121473 | 0.37 |
| TotalSeed | CWSeedNo | 0.1442 | 0.329899 | 1.5233 | 19 | -0.1186911 | 0.6666465 | 2.32 |
| TotalSeed | CCWSeedNo | 0.2396 | 0.268306 | 1.214 | 19 | -0.1847835 | 0.6273323 | 1.474 |
| TotalSeed | OntoDir | 0.5343 | 0.143707 | 0.63297 | 19 | -0.3070267 | 0.5417834 | 0.4007 |
| TotalSeed | AxisWidth | 0.3312 | 0.222997 | 0.99713 | 19 | -0.2309192 | 0.5971949 | 0.994 |
| TotalSeed | AxisLength | 0.1038 | 0.364914 | 1.7084 | 19 | -0.0792583 | 0.688191 | 2.919 |
| CWNo | Circumference | 0.7726 | -0.0671 | -0.29315 | 19 | -0.4847476 | 0.3754602 | 0.086 |
| CWNo | Length | 0.2085 | -0.28618 | -1.3019 | 19 | -0.6389359 | 0.1660116 | 1.695 |
| CWNo | Width | 0.6034 | -0.12031 | -0.52827 | 19 | -0.5247462 | 0.3284313 | 0.279 |
| CWNo | CCWNo | 0.0192 | -0.50624 | -2.5588 | 19 | -0.7697154 | -0.095402 | 6.547 |
| CWNo | CWDeg | 0.05984 | 0.417273 | 2.0014 | 19 | -0.7193775 | 0.0175805 | 4.006 |
| CWNo | CCWDeg | 0.5489 | 0.138674 | 0.61036 | 19 | -0.311671 | 0.538145 | 0.373 |
| CWNo | CWSeedNo | 0.001636 | 0.643835 | 3.6677 | 19 | -0.8416089 | -0.293807 | 13.45 |
| CWNo | CCWSeedNo | 0.2541 | 0.260498 | 1.1761 | 19 | -0.1928788 | 0.6222145 | 1.383 |
| CWNo | AxisWidth | 0.4324 | -0.18098 | -0.8021 | 19 | -0.5682664 | 0.2719582 | 0.643 |
| CWNo | AxisLength | 0.07661 | -0.39471 | -1.8725 | 19 | -0.7060846 | 0.0445741 | 3.506 |
| CCWNo | Circumference | 0.9803 | -0.00574 | -0.02502 | 19 | -0.4363461 | 0.4270043 | 0.001 |
| CCWNo | Length | 0.9976 | 0.000702 | 0.003059 | 19 | -0.4311156 | 0.4322576 | 0 |
| CCWNo | Width | 0.9685 | 0.009192 | 0.040069 | 19 | -0.4241779 | 0.4391363 | 0.002 |
| CCWNo | CWNo | 0.0192 | -0.50624 | -2.5588 | 19 | -0.7697154 | -0.095402 | 6.547 |
| CCWNo | CWDeg | 0.5351 | 0.143432 | 0.63174 | 19 | -0.3072811 | 0.5415849 | 0.399 |
| CCWNo | CCWDeg | 0.4393 | 0.178335 | 0.79001 | 19 | -0.2744828 | 0.5664163 | 0.624 |
| CCWNo | CWSeedNo | 0.1563 | 0.32078 | 1.4763 | 19 | -0.1287337 | 0.6609417 | 2.179 |
| CCWNo | CCWSeedNo | 0.755 | -0.07244 | -0.3166 | 19 | -0.4888423 | 0.3708411 | 0.1 |
| CCWNo | AxisWidth | 0.9091 | 0.026523 | 0.11565 | 19 | -0.4098566 | 0.4530227 | 0.013 |
| CCWNo | AxisLength | 0.9645 | -0.01036 | -0.04515 | 19 | -0.4400762 | 0.423222 | 0.002 |
| CCWNo | OntoDir | 0.531 | 0.144946 | 0.63855 | 19 | -0.3058801 | 0.5426768 | 0.4077 |
| CWDeg | Circumference | 0.2521 | -0.26154 | -1.1811 | 19 | -0.6229007 | 0.1917997 | 1.395 |
| CWDeg | Length | 0.6057 | -0.11958 | -0.52499 | 19 | -0.5242041 | 0.3290982 | 0.276 |
| CWDeg | Width | 0.1903 | -0.29751 | -1.3583 | 19 | -0.6462017 | 0.1539525 | 1.845 |
| CWDeg | CWNo | 0.05984 | 0.417273 | 2.0014 | 19 | -0.7193775 | 0.0175805 | 4.006 |
| CWDeg | CCWNo | 0.5351 | 0.143432 | 0.63174 | 19 | -0.3072811 | 0.5415849 | 0.399 |
| CWDeg | CCWDeg | 0.1007 | -0.36801 | -1.7252 | 19 | -0.6900715 | 0.0756987 | 2.976 |
| CWDeg | CWSeedNo | 1.27E-05 | 0.801258 | 5.8374 | 19 | 0.5650003 | 0.9160796 | 34.08 |
| CWDeg | CCWSeedNo | 0.3306 | -0.22328 | -0.99846 | 19 | -0.5973857 | 0.2306383 | 0.997 |
| CWDeg | AxisWidth | 0.5833 | 0.126991 | 0.55806 | 19 | -0.322368 | 0.5296427 | 0.311 |
| CWDeg | AxisLength | 0.5608 | 0.134593 | 0.59206 | 19 | -0.3154206 | 0.5351843 | 0.351 |
| CCWDeg | Circumference | 0.5346 | 0.143617 | 0.63257 | 19 | -0.3071095 | 0.5417188 | 0.4 |
| CCWDeg | Length | 0.2706 | 0.251943 | 0.63257 | 19 | -0.2016783 | 0.6165713 | 1.288 |
| CCWDeg | Width | 0.4273 | 0.182945 | 0.81113 | 19 | -0.2700708 | 0.569644 | 0.658 |
| CCWDeg | CWNo | 0.5489 | 0.138674 | 0.61036 | 19 | -0.311671 | 0.538145 | 0.373 |
| CCWDeg | CCWNo | 0.4393 | 0.178335 | 0.79001 | 19 | -0.2744828 | 0.5664163 | 0.624 |
| CCWDeg | CWDeg | 0.1007 | -0.36801 | -1.7252 | 19 | -0.6900715 | 0.0756987 | 2.976 |
| CCWDeg | CWSeedNo | 0.1791 | -0.30484 | -1.3952 | 19 | -0.6508754 | 0.1460675 | 1.947 |
| CCWDeg | CCWSeedNo | 0.000401 | 0.700945 | 4.2839 | 19 | 0.386082 | 0.8695236 | 18.35 |
| CCWDeg | AxisWidth | 0.03974 | -0.45186 | -2.2079 | 19 | -0.7393328 | -0.025065 | 4.875 |
| CCWDeg | AxisLength | 0.7689 | 0.068231 | 0.29811 | 19 | -0.3744861 | 0.4856143 | 0.089 |
| CWSeedNo | Circumference | 0.7356 | -0.07838 | -0.3427 | 19 | -0.4933724 | 0.3656807 | 0.117 |
| CWSeedNo | Length | 0.4764 | 0.164416 | 0.72656 | 19 | -0.2876898 | 0.5565977 | 0.528 |
| CWSeedNo | Width | 0.662 | -0.10135 | -0.44404 | 19 | -0.5106902 | 0.3454544 | 0.197 |
| CWSeedNo | CWNo | 0.001636 | 0.643835 | 3.6677 | 19 | -0.8416089 | -0.293807 | 13.45 |
| CWSeedNo | CCWNo | 0.1563 | 0.32078 | 1.4763 | 19 | -0.1287337 | 0.6609417 | 2.179 |
| CWSeedNo | CWDeg | 1.27E-05 | 0.801258 | 5.8374 | 19 | 0.5650003 | 0.9160796 | 34.08 |
| CWSeedNo | CCWDeg | 0.1791 | -0.30484 | -1.3952 | 19 | -0.6508754 | 0.1460675 | 1.947 |
| CWSeedNo | CCWSeedNo | 0.165 | -0.31451 | -1.4442 | 19 | -0.656995 | 0.1355877 | 2.086 |
| CWSeedNo | AxisWidth | 0.2508 | 0.262232 | 1.1845 | 19 | -0.1910865 | 0.6233536 | 1.403 |
| CWSeedNo | AxisLength | 0.04942 | 0.433844 | 2.0989 | 19 | 0.00265407 | 0.7290001 | 4.405 |
| CCWSeedNo | Circumference | 0.2695 | 0.252477 | 1.1374 | 19 | -0.2011312 | 0.6169246 | 1.294 |
| CCWSeedNo | Length | 0.6989 | 0.089734 | 0.39272 | 19 | -0.355733 | 0.5019756 | 0.154 |
| CCWSeedNo | Width | 0.2682 | 0.253145 | 1.1406 | 19 | -0.2004462 | 0.6173666 | 1.301 |
| CCWSeedNo | CWNo | 0.2541 | 0.260498 | 1.1761 | 19 | -0.1928788 | 0.6222145 | 1.383 |
| CCWSeedNo | CWDeg | 0.3306 | -0.22328 | -0.99846 | 19 | -0.5973857 | 0.2306383 | 0.997 |
| CCWSeedNo | CCWDeg | 0.000401 | 0.700945 | 4.2839 | 19 | 0.386082 | 0.8695236 | 18.35 |
| CCWSeedNo | CWSeedNo | 0.165 | -0.31451 | -1.4442 | 19 | -0.656995 | 0.1355877 | 2.086 |
| CCWSeedNo | AxisWidth | 0.06993 | -0.40319 | -1.9205 | 19 | -0.7111102 | 0.0344961 | 3.688 |
| CCWSeedNo | AxisLength | 0.744 | -0.0758 | -0.33136 | 19 | -0.4914074 | 0.3679256 | 0.11 |
| OntoDir | Circumference | 0.6795 | 0.095805 | 0.41953 | 19 | -0.3503726 | 0.5065421 | 0.176 |
| OntoDir | Length | 0.6102 | 0.11807 | 0.51828 | 19 | -0.3304601 | 0.523095 | 0.2686 |
| OntoDir | Width | 0.4468 | 0.175474 | 0.77693 | 19 | -0.2772118 | 0.5644068 | 0.6036 |
| OntoDir | CWNo | 0.9137 | 0.025187 | 0.10982 | 19 | -0.4109681 | 0.4519597 | 0.01206 |
| OntoDir | CCWNo | 0.5307 | 0.144946 | 0.63855 | 19 | -0.3058801 | 0.5426768 | 0.4077 |
| OntoDir | CWDeg | 0.5049 | 0.15406 | 0.67965 | 19 | -0.297406 | 0.5492205 | 0.4619 |
| OntoDir | CCWDeg | 0.1281 | 0.342898 | 1.5911 | 19 | -0.1042155 | 0.6747109 | 2.532 |
| OntoDir | CWSeedNo | 0.7151 | 0.084691 | 0.37049 | 19 | -0.360163 | 0.4981652 | 0.1373 |
| OntoDir | CCWSeedNo | 0.08549 | 0.384232 | 1.8141 | 19 | -0.0568913 | 0.6998381 | 3.291 |
| Width | AxisWidth | 0.09396 | 0.374976 | 1.7631 | 19 | -0.0676636 | 0.6942784 | 3.109 |
| Width | AxisLength | 0.001267 | 0.655129 | 3.7797 | 19 | 0.3115521 | 0.8472144 | 14.29 |
| Length | AxisWidth | 0.08773 | 0.381719 | 1.8002 | 19 | -0.0598263 | 0.6983323 | 3.241 |
| Length | AxisLength | 1.55E-06 | 0.843633 | 6.8489 | 19 | 0.6479034 | 0.9348583 | 46.91 |
| Circumference | AxisWidth | 0.05478 | 0.425014 | 2.0466 | 19 | -0.0081724 | 0.723887 | 4.189 |
| Circumference | AxisLength | 0.001979 | 0.635112 | 3.584 | 19 | 0.2802647 | 0.8372501 | 12.85 |
| Circumference | Length | 7.50E-06 | 0.812952 | 6.0852 | 19 | 0.5874116 | 0.9213126 | 37.03 |

Supplemental Table S2. Pearson correlation coefficients between measured variables used in the multiple linear regression models. CWSeedNo: number of seeds in the segmented CW parastichy. CCWSeedNo: number of seeds in the segmented CCW parastichy. CWDeg: degree of rotation of the CW parastichy. CCWDeg: degree of rotation of the CCW parastichy. AxisWidth: width of cone axis. AxisLength: length of cone axis. CWNo: number of clockwise parastichies. CCWNo: number of counterclockwise parastichies. PseudoNo: number of pseudoparastichies. TotalSeed: number of bract/scale complexes in cone. Length: cone length. Width: cone width. Circumference: cone circumference. OntoDir: direction of ontogenetic growth.

|  | CWSeedNo | CCWSeedNo | CWDeg | CCWDeg | AxisWidth | AxisLength | CWNo | CCWNo |
| --- | --- | --- | --- | --- | --- | --- | --- | --- |
| CWSeedNo | 1 | -0.30365 | 0.660217 | 0.049177 | -0.16503 | 0.365609 | 0.458847 | 0.268388 |
| CCWSeedNo | -0.30365 | 1 | 0.566125 | 0.83591 | 0.155791 | -0.22165 | -0.33373 | -0.49664 |
| CWDeg | 0.660217 | 0.566125 | 1 | -0.53025 | -0.20801 | 0.16941 | 0.215345 | 0.052094 |
| CCWDeg | 0.049177 | 0.83591 | -0.53025 | 1 | -0.53107 | 0.412194 | 0.291603 | 0.65253 |
| AxisWidth | -0.16503 | 0.155791 | -0.20801 | -0.53107 | 1 | 0.448517 | 0.048358 | 0.363835 |
| AxisLength | 0.365609 | -0.22165 | 0.16941 | 0.412194 | 0.448517 | 1 | 0.054556 | -0.36625 |
| CWNo | 0.458847 | -0.33373 | 0.215345 | 0.291603 | 0.048358 | 0.054556 | 1 | -0.40687 |
| CCWNo | 0.268388 | -0.49664 | 0.052094 | 0.65253 | 0.363835 | -0.36625 | -0.40687 | 1 |
| PseudoNo | 0.05202 | 0.170536 | 0.094172 | -0.13658 | -0.07733 | -0.35289 | 0.191213 | 0.232947 |
| TotalSeed | 0.599812 | 0.630075 | -0.72763 | -0.54307 | -0.17272 | 0.226715 | 0.313506 | 0.090404 |
| Length | 0.268671 | -0.41877 | 0.037677 | 0.579941 | 0.296678 | 0.094922 | -0.13363 | -0.53384 |
| Width | -0.44628 | 0.139222 | -0.18498 | -0.42247 | -0.51929 | 0.510866 | -0.33455 | 0.271 |
| Circumference | 0.307187 | 0.037585 | 0.199082 | 0.232065 | 0.501977 | -0.45042 | 0.391478 | -0.04678 |
| OntoDir | 0.281125 | -0.09381 | 0.292752 | 0.414085 | 0.520703 | -0.47903 | 0.208914 | -0.16295 |

|  | PseudoNo | Total Seed | Length | Width | Circumference | OntoDir |
| --- | --- | --- | --- | --- | --- | --- |
| CWSeedNo | 0.05201995 | 0.599812 | 0.268671 | -0.44628 | 0.307187 | 0.281125 |
| CCWSeedNo | 0.17053556 | 0.630075 | -0.41877 | 0.139222 | 0.037585 | -0.09381 |
| CWDeg | 0.09417245 | -0.72763 | 0.037677 | -0.18498 | 0.199082 | 0.292752 |
| CCWDeg | -0.13658261 | -0.54307 | 0.579941 | -0.42247 | 0.232065 | 0.414085 |
| AxisWidth | -0.07733001 | -0.17272 | 0.296678 | -0.51929 | 0.501977 | 0.520703 |
| AxisLength | -0.35289333 | 0.226715 | 0.094922 | 0.510866 | -0.45042 | -0.47903 |
| CWNo | 0.19121336 | 0.313506 | -0.13363 | -0.33455 | 0.391478 | 0.208914 |
| CCWNo | 0.23294717 | 0.090404 | -0.53384 | 0.271 | -0.04678 | -0.16295 |
| PseudoNo | 1 | 0.229551 | 0.348232 | 0.163528 | -0.27825 | -0.14432 |
| TotalSeed | 0.22955142 | 1 | -0.03277 | -0.0818 | 0.145707 | 0.187994 |
| Length | 0.34823216 | -0.03277 | 1 | 0.431874 | -0.13902 | -0.28952 |
| Width | 0.1635277 | -0.0818 | 0.431874 | 1 | 0.924695 | 0.756559 |
| Circumference | -0.27825057 | 0.145707 | -0.13902 | 0.924695 | 1 | -0.71898 |
| OntoDir | -0.1443229 | 0.187994 | -0.28952 | 0.756559 | -0.71898 | 1 |

Supplemental Table S3. Summary of multiple linear regression models. Regression coefficients (Estimates) with standard errors (SE), *t-*statistic, and *p-*value are presented for each model. Model fit statistics include Multiple R², Adjusted R², *F-*statistic (F), degrees of freedom (df), and model *p-*value. Correlation coefficients for individual interactions are also given. CWSeedNo: number of seeds in the segmented CW parastichy. CCWSeedNo: number of seeds in the segmented CCW parastichy. CWDeg: degree of rotation of the CW parastichy. CCWDeg: degree of rotation of the CCW parastichy. AxisWidth: width of cone axis. AxisLength: length of cone axis. CWNo: number of clockwise parastichies. CCWNo: number of counterclockwise parastichies. PseudoNo: number of pseudoparastichies. TotalSeed: number of bract/scale complexes in cone. Length: cone length. Width: cone width. Circumference: cone circumference. OntoDir: direction of ontogenetic growth.

Model 1: TotalSeed ~ Circumference + Length + Width

| Variable x | Variable y_1_ | | Variable y_2_ | Variable y_3_ | | Estimate | | SE | | | t | | | *p-*value |
| --- | --- | --- | --- | --- | --- | --- | --- | --- | --- | --- | --- | --- | --- | --- |
| TotalSeed | Circumference | |  |  | | -143.77728 | | 264.74784 | | | -0.543 | | | 0.596 |
| TotalSeed | Length | |  |  | | -33.65405 | | 53.24328 | | | -0.632 | | | 0.538 |
| TotalSeed | Width | |  |  | | -74.21889 | | 81.8815 | | | -0.906 | | | 0.381 |
| TotalSeed | Circumference | | Length |  | | 1.81802 | | 3.98866 | | | 0.456 | | | 0.656 |
| TotalSeed | Circumference | | Width |  | | 3.66035 | | 3.59297 | | | 1.019 | | | 0.327 |
| TotalSeed | Length | | Width |  | | 0.83007 | | 1.15442 | | | 0.719 | | | 0.485 |
| TotalSeed | Circumference | | Length | Width | | -0.04278 | | 0.0487 | | | -0.878 | | | 0.396 |
|  |  | |  |  | |  | |  | | |  | | |  |
| Model Fit Statistics | | |  |  | |  | |  | | |  | | |  |
| Multiple R^2^ | 0.3041 | |  |  | |  | |  | | |  | | |  |
| Adjusted R^2^ | -0.07061 | |  |  | |  | |  | | |  | | |  |
| *F-*statistic | 0.8116 | |  |  | |  | |  | | |  | | |  |
| df | 7, 13 | |  |  | |  | |  | | |  | | |  |
| *p-*value | 0.5934 | |  |  | |  | |  | | |  | | |  |
|  |  | |  |  | |  | |  | | |  | | |  |
|  |  | |  |  | |  | |  | | |  | | |  |
|  |  | |  |  | |  | |  | | |  | | |  |
|  |  | |  |  | |  | |  | | |  | | |  |
|  |  | |  |  | |  | |  | | |  | | |  |
|  |  | |  |  | |  | |  | | |  | | |  |
| Model 2: TotalSeed ~ CWNo + CCWNo | | | | |  | |  | | |  | | |  | |
| Variable x | Variable y_1_ | | Variable y_2_ | Variable y_3_ | | Estimate | | SE | | | *t-*value | | | *p-*value |
| TotalSeed | CWNo | |  |  | | 127.918 | | 75.54 | | | 1.693 | | | 0.109 |
| TotalSeed | CCWNo | |  |  | | 126.933 | | 74.635 | | | 1.701 | | | 0.107 |
| TotalSeed | CWNo | | CCWNo |  | | -9.559 | | 5.546 | | | -1.723 | | | 0.103 |
|  |  | |  |  | |  | |  | | |  | | |  |
| Model Fit Statistics | | |  |  | |  | |  | | |  | | |  |
| Multiple R^2^ | 0.1607 | |  |  | |  | |  | | |  | | |  |
| Adjusted R^2^ | 0.01253 | |  |  | |  | |  | | |  | | |  |
| *F-*statistic | 1.085 | |  |  | |  | |  | | |  | | |  |
| df | 3, 17 | |  |  | |  | |  | | |  | | |  |
| *p-*value | 0.3822 | |  |  | |  | |  | | |  | | |  |
|  |  | |  |  | |  | |  | | |  | | |  |
|  |  | |  |  | |  | |  | | |  | | |  |
| Model 3: TotalSeed ~ CWDeg + CCWDeg | | | | |  | |  | | |  | | |  | |
| Variable x | Variable y_1_ | | Variable y_2_ | Variable y_3_ | | Estimate | | SE | | | *t-*value | | | *p-*value |
| TotalSeed | CWDeg | |  |  | | -0.357011 | | 0.549542 | | | -0.65 | | | 0.525 |
| TotalSeed | CCWDeg | |  |  | | -0.394334 | | 0.528681 | | | -0.746 | | | 0.466 |
| TotalSeed | CWDeg | | CCWDeg |  | | 0.001004 | | 0.001536 | | | 0.654 | | | 0.522 |
|  |  | |  |  | |  | |  | | |  | | |  |
| Model Fit Statistics | | |  |  | |  | |  | | |  | | |  |
| Multiple R^2^ | 0.04321 | |  |  | |  | |  | | |  | | |  |
| Adjusted R^2^ | -0.1256 | |  |  | |  | |  | | |  | | |  |
| *F-*statistic | 0.2559 | |  |  | |  | |  | | |  | | |  |
| df | 3, 17 | |  |  | |  | |  | | |  | | |  |
| *p-*value | 0.8561 | |  |  | |  | |  | | |  | | |  |
|  |  | |  |  | |  | |  | | |  | | |  |
|  |  | |  |  | |  | |  | | |  | | |  |
|  |  | |  |  | |  | |  | | |  | | |  |
|  |  | |  |  | |  | |  | | |  | | |  |
| Model 4: TotalSeed ~ CWSeedNo + CCWSeedNo | | | | | |  | | |  | | |  | | |
| Variable x | Variable y_1_ | | Variable y_2_ | Variable y_3_ | | Estimate | | SE | | | *t-*value | | | *p-*value |
| TotalSeed | CWSeedNo | |  |  | | 6.9704 | | 13.4466 | | | 0.518 | | | 0.611 |
| TotalSeed | CCWSeedNo | |  |  | | 6.6484 | | 13.1532 | | | 0.505 | | | 0.62 |
| TotalSeed | CWSeedNo | | CCWSeedNo |  | | -0.0934 | | 0.446 | | | -0.209 | | | 0.837 |
|  |  | |  |  | |  | |  | | |  | | |  |
| Model Fit Statistics | | |  |  | |  | |  | | |  | | |  |
| Multiple R^2^ | 0.2644 | |  |  | |  | |  | | |  | | |  |
| Adjusted R^2^ | | 0.1345 |  | |  | |  | | |  | | |  | |
| *F-*statistic | 2.036 | |  |  | |  | |  | | |  | | |  |
| df | 3, 17 | |  |  | |  | |  | | |  | | |  |
| *p-*value | 0.1469 | |  |  | |  | |  | | |  | | |  |
|  |  | |  |  | |  | |  | | |  | | |  |
|  |  | |  |  | |  | |  | | |  | | |  |
| Model 5: TotalSeed ~ CWSeedNo + CWDeg | | | |  | |  | |  | | |  | | |  |
| Variable x | Variable y_1_ | | Variable y_2_ | Variable y_3_ | | Estimate | | SE | | | *t-*value | | | *p-*value |
| TotalSeed | CWSeedNo | |  |  | | 2.93254 | | 5.3776 | | | 0.545 | | | 0.5926 |
| TotalSeed | CWDeg | |  |  | | -0.70071 | | 0.4602 | | | -1.523 | | | 0.1462 |
| TotalSeed | CWSeedNo | | CWDeg |  | | 0.01286 | | 0.01231 | | | 1.045 | | | 0.3106 |
|  |  | |  |  | |  | |  | | |  | | |  |
| Model Fit Statistics | | |  |  | |  | |  | | |  | | |  |
| Multiple R^2^ | 0.2917 | |  |  | |  | |  | | |  | | |  |
| Adjusted R^2^ | | 0.1667 |  | |  | |  | | |  | | |  | |
| *F-*statistic | 2.333 | |  |  | |  | |  | | |  | | |  |
| df | 3, 17 | |  |  | |  | |  | | |  | | |  |
| *p-*value | 0.1104 | |  |  | |  | |  | | |  | | |  |
|  |  | |  |  | |  | |  | | |  | | |  |
|  |  | |  |  | |  | |  | | |  | | |  |
|  |  | |  |  | |  | |  | | |  | | |  |
|  |  | |  |  | |  | |  | | |  | | |  |
| Model 6: TotalSeed ~ CWNo + CWSeedNo + CWDeg | | | | | |  | |  | | |  | | |  |
| Variable x | Variable y_1_ | | Variable y_2_ | Variable y_3_ | | Estimate | | SE | | | *t-*value | | | *p-*value |
| TotalSeed | CWNo | |  |  | | -41.035202 | | 46.068679 | | | -0.891 | | | 0.3893 |
| TotalSeed | CWSeedNo | |  |  | | -6.666302 | | 27.468305 | | | -0.243 | | | 0.812 |
| TotalSeed | CWDeg | |  |  | | -3.197219 | | 2.135133 | | | -1.497 | | | 0.1582 |
| TotalSeed | CWNo | | CWSeedNo |  | | 1.366076 | | 1.554652 | | | 0.879 | | | 0.3955 |
| TotalSeed | CWNo | | CWDeg |  | | 0.238493 | | 0.134163 | | | 1.778 | | | 0.0989 |
| TotalSeed | CWSeedNo | | CWDeg |  | | 0.091125 | | 0.057225 | | | 1.592 | | | 0.1353 |
| TotalSeed | CWNo | | CWSeedNo | CWDeg | | -0.007405 | | 0.003629 | | | -2.04 | | | 0.0622 |
|  |  | |  |  | |  | |  | | |  | | |  |
| Model Fit Statistics | | |  |  | |  | |  | | |  | | |  |
| Multiple R^2^ | 0.6094 | |  |  | |  | |  | | |  | | |  |
| Adjusted R^2^ | | 0.3991 |  | |  | |  | | |  | | |  | |
| *F-*statistic | 0.7806408 | |  |  | |  | |  | | |  | | |  |
| df | 7, 13 | |  |  | |  | |  | | |  | | |  |
| *p-*value | 0.04649 | |  |  | |  | |  | | |  | | |  |
|  |  | |  |  | |  | |  | | |  | | |  |
|  | | | | | |  | |  | | |  | | |  |
|  | | | | | |  | |  | | |  | | |  |
|  | | | | | |  | |  | | |  | | |  |
|  | | | | | |  | |  | | |  | | |  |
|  | | | | | |  | |  | | |  | | |  |
|  | | | | | |  | |  | | |  | | |  |
|  | | | | | |  | |  | | |  | | |  |
|  | | | | | |  | |  | | |  | | |  |
|  | | | | | |  | |  | | |  | | |  |
|  | | | | | |  | |  | | |  | | |  |
|  | | | | | |  | |  | | |  | | |  |
|  | | | | | |  | |  | | |  | | |  |
|  | | | | | |  | |  | | |  | | |  |
| Model 7: TotalSeed ~ CCWNo + CCWSeedNo + CCWDeg | | | | | |  | |  | | |  | | |  |
| Variable x | Variable y_1_ | | Variable y_2_ | Variable y_3_ | | Estimate | | SE | | | *t-*value | | | *p-*value |
| TotalSeed | CCWNo | |  |  | | 137.60 | | 89.28 | | | 1.541 | | | 0.147 |
| TotalSeed | CCWSeedNo | |  |  | | 82.08 | | 48.17 | | | 1.704 | | | 0.112 |
| TotalSeed | CCWDeg | |  |  | | 7.07 | | 5.27 | | | 1.342 | | | 0.203 |
| TotalSeed | CCWNo | | CCWSeedNo |  | | -3.62 | | 2.43 | | | -1.492 | | | 0.159 |
| TotalSeed | CCWNo | | CCWDeg |  | | -0.35 | | 0.26 | | | -1.358 | | | 0.198 |
| TotalSeed | CCWSeedNo | | CCWDeg |  | | -0.19 | | 0.14 | | | -1.392 | | | 0.187 |
| TotalSeed | CCWNo | | CCWSeedNo | CCWDeg | | 0.01 | | 0.01 | | | 1.358 | | | 0.198 |
|  |  | |  |  | |  | |  | | |  | | |  |
| Model Fit Statistics | | |  |  | |  | |  | | |  | | |  |
| Multiple R^2^ | 0.4628 | |  |  | |  | |  | | |  | | |  |
| Adjusted R^2^ | | 0.1735 |  | |  | |  | | |  | | |  | |
| *F-*statistic | 1.6 | |  |  | |  | |  | | |  | | |  |
| df | 7, 13 | |  |  | |  | |  | | |  | | |  |
| *p-*value | 0.2203 | |  |  | |  | |  | | |  | | |  |
|  |  | |  |  | |  | |  | | |  | | |  |
|  |  | |  |  | |  | |  | | |  | | |  |
|  |  | |  |  | |  | |  | | |  | | |  |
|  |  | |  |  | |  | |  | | |  | | |  |
|  |  | |  |  | |  | |  | | |  | | |  |
|  |  | |  |  | |  | |  | | |  | | |  |
|  |  | |  |  | |  | |  | | |  | | |  |
|  |  | |  |  | |  | |  | | |  | | |  |
|  |  | |  |  | |  | |  | | |  | | |  |
|  |  | |  |  | |  | |  | | |  | | |  |
|  |  | |  |  | |  | |  | | |  | | |  |
|  |  | |  |  | |  | |  | | |  | | |  |
|  |  | |  |  | |  | |  | | |  | | |  |
|  |  | |  |  | |  | |  | | |  | | |  |
| Model 8: TotalSeed ~ AxisLength + AxisWidth + CWSeedNo + CCWSeedNo | | | | | | | |  | | |  | | |  |
| Variable x | Variable y_1_ | | Variable y_2_ | Variable y_3_ | | Estimate | | SE | | | *t-*value | | | *p-*value |
| TotalSeed | AxisLength | |  |  | | -2169.4808 | | 2187.1779 | | | -0.992 | | | 0.367 |
| TotalSeed | AxisWidth | |  |  | | -3603.9116 | | 3973.8094 | | | -0.907 | | | 0.406 |
| TotalSeed | CWSeedNo | |  |  | | -3357.86 | | 3314.7522 | | | -1.013 | | | 0.358 |
| TotalSeed | CCWSeedNo | |  |  | | -2848.6708 | | 2949.8792 | | | -0.966 | | | 0.379 |
| TotalSeed | AxisLength | | AxisWidth |  | | 84.2658 | | 97.2725 | | | 0.866 | | | 0.426 |
| TotalSeed | AxisLength | | CWSeedNo |  | | 81.2554 | | 80.0953 | | | 1.014 | | | 0.357 |
| TotalSeed | AxisWidth | | CWSeedNo |  | | 139.1585 | | 152.0983 | | | 0.915 | | | 0.402 |
| TotalSeed | AxisLength | | CCWSeedNo |  | | 67.9703 | | 71.794 | | | 0.947 | | | 0.387 |
| TotalSeed | AxisWidth | | CCWSeedNo |  | | 114.3304 | | 133.1391 | | | 0.859 | | | 0.43 |
| TotalSeed | CWSeedNo | | CCWSeedNo |  | | 109.1038 | | 112.2951 | | | 0.972 | | | 0.376 |
| TotalSeed | AxisLength | | AxisWidth | CWSeedNo | | -3.2753 | | 3.6592 | | | -0.895 | | | 0.412 |
| TotalSeed | AxisLength | | AxisWidth | CCWSeedNo | | -2.6256 | | 3.2282 | | | -0.813 | | | 0.453 |
| TotalSeed | AxisLength | | CWSeedNo | CCWSeedNo | | -2.6145 | | 2.6917 | | | -0.971 | | | 0.376 |
| TotalSeed | AxisWidth | | CWSeedNo | CCWSeedNo | | -4.5505 | | 5.2269 | | | -0.871 | | | 0.424 |
|  |  | |  |  | |  | |  | | |  | | |  |
| Model Fit Statistics | | |  |  | |  | |  | | |  | | |  |
| Multiple R^2^ | 0.792 | |  |  | |  | |  | | |  | | |  |
| Adjusted R^2^ | | 0.168 |  | |  | |  | | |  | | |  | |
| *F-*statistic | | 1.27 |  | |  | |  | | |  | | |  | |
| df | 15, 5 | |  |  | |  | |  | | |  | | |  |
| *p-*value | 0.426 | |  |  | |  | |  | | |  | | |  |
|  |  | |  |  | |  | |  | | |  | | |  |
|  |  | |  |  | |  | |  | | |  | | |  |
|  |  | |  |  | |  | |  | | |  | | |  |
|  |  | |  |  | |  | |  | | |  | | |  |
|  |  | |  |  | |  | |  | | |  | | |  |
|  |  | |  |  | |  | |  | | |  | | |  |
|  |  | |  |  | |  | |  | | |  | | |  |
| Model 9: TotalSeed ~ Circumference + AxisLength + CWSeedNo | | | | | | | |  | | |  | | |  |
| Variable x | Variable y_1_ | | Variable y_2_ | Variable y_3_ | | Estimate | | SE | | | *t-*value | | | *p-*value |
| TotalSeed | Circumference | |  |  | | -152.33541 | | 58.62561 | | | -2.598 | | | 0.02207 |
| TotalSeed | AxisLength | |  |  | | -107.4117 | | 33.69996 | | | -3.187 | | | 0.00714 |
| TotalSeed | CWSeedNo | |  |  | | -100.38622 | | 45.88227 | | | -2.188 | | | 0.04754 |
| TotalSeed | Circumference | | AxisLength |  | | 5.27987 | | 1.75644 | | | 3.006 | | | 0.01012 |
| TotalSeed | Circumference | | CWSeedNo |  | | 5.39818 | | 2.43064 | | | 2.221 | | | 0.04475 |
| TotalSeed | AxisLength | | CWSeedNo |  | | 3.20858 | | 1.30665 | | | 2.456 | | | 0.02891 |
| TotalSeed | Circumference | | AxisLength | CWSeedNo | | -0.16112 | | 0.06763 | | | -2.382 | | | 0.03316 |
|  |  | |  |  | |  | |  | | |  | | |  |
| Model Fit Statistics | | |  |  | |  | |  | | |  | | |  |
| Multiple R^2^ | 0.713 | |  |  | |  | |  | | |  | | |  |
| Adjusted R^2^ | | 0.5584 |  | |  | |  | | |  | | |  | |
| *F-*statistic | 4.613 | |  |  | |  | |  | | |  | | |  |
| df | 7, 13 | |  |  | |  | |  | | |  | | |  |
| *p-*value | 0.008583 | |  |  | |  | |  | | |  | | |  |
|  |  | |  |  | |  | |  | | |  | | |  |
|  |  | |  |  | |  | |  | | |  | | |  |
|  |  | |  |  | |  | |  | | |  | | |  |
|  |  | |  |  | |  | |  | | |  | | |  |
|  |  | |  |  | |  | |  | | |  | | |  |
|  |  | |  |  | |  | |  | | |  | | |  |
|  |  | |  |  | |  | |  | | |  | | |  |
|  |  | |  |  | |  | |  | | |  | | |  |
|  |  | |  |  | |  | |  | | |  | | |  |
|  |  | |  |  | |  | |  | | |  | | |  |
|  |  | |  |  | |  | |  | | |  | | |  |
|  |  | |  |  | |  | |  | | |  | | |  |
|  |  | |  |  | |  | |  | | |  | | |  |
|  |  | |  |  | |  | |  | | |  | | |  |
| Model 10: TotalSeed ~ CWSeedNo + CWDeg + OntoDir + CWNo | | | | | | | |  | | |  | | |  |
| Variable x | Variable y_1_ | | Variable y_2_ | Variable y_3_ | | Estimate | | SE | | | *t-*value | | | *p-*value |
| TotalSeed | CWSeedNo | |  |  | | -4.51E+01 | | 1.20E+01 | | | -0.71 | | | 0.50926 |
| TotalSeed | CWDeg | |  |  | | -2.07E+00 | | 1.01E+00 | | | -2.055 | | | 0.09499 |
| TotalSeed | OntoDir | |  |  | | 3.57E+03 | | 2.75E+03 | | | 1.298 | | | 0.2509 |
| TotalSeed | CWNo | |  |  | | -6.80E+01 | | 2.03E+01 | | | -3.341 | | | 0.02052 |
| TotalSeed | CWSeedNo | | CWDeg |  | | 1.05E-01 | | 2.84E-02 | | | 3.689 | | | 0.01416 |
| TotalSeed | CWSeedNo | | OntoDir |  | | -6.95E+01 | | 9.79E+01 | | | -3.757 | | | 0.0132 |
| TotalSeed | CWDeg | | OntoDir |  | | -1.19E+01 | | 3.37E+00 | | | -3.539 | | | 0.01657 |
| TotalSeed | CWSeedNo | | CWNo |  | | 4.10E+00 | | 7.48E-01 | | | 5.488 | | | 0.00274 |
| TotalSeed | CWDeg | | CWNo |  | | 1.42E-01 | | 6.53E-02 | | | 2.178 | | | 0.08137 |
| TotalSeed | OntoDir | | CWNo |  | | -2.10E+02 | | 1.20E+02 | | | -1.749 | | | 0.14075 |
| TotalSeed | CWSeedNo | | CWDeg | OntoDir | | 2.91E-01 | | 1.15E-01 | | | 2.536 | | | 0.05215 |
| TotalSeed | CWSeedNo | | OntoDir | CWNo | | -7.74E-03 | | 1.88E-03 | | | -4.123 | | | 0.00915 |
| TotalSeed | CWSeedNo | | CWDeg | CWNo | | 3.61E+00 | | 4.15E+00 | | | 0.871 | | | 0.42367 |
| TotalSeed | CWDeg | | OntoDir | CWNo | | 8.41E-01 | | 1.95E-01 | | | 4.307 | | | 0.00767 |
|  |  | |  |  | |  | |  | | |  | | |  |
| Model Fit Statistics | | |  |  | |  | |  | | |  | | |  |
| Multiple R^2^ | 0.983 | |  |  | |  | |  | | |  | | |  |
| Adjusted R^2^ | | 0.932 |  | |  | |  | | |  | | |  | |
| *F-*statistic | | 19.4 |  | |  | |  | | |  | | |  | |
| df | 15, 5 | |  |  | |  | |  | | |  | | |  |
| *p-*value | 0.00201 | |  |  | |  | |  | | |  | | |  |
|  |  | |  |  | |  | |  | | |  | | |  |
|  |  | |  |  | |  | |  | | |  | | |  |
|  |  | |  |  | |  | |  | | |  | | |  |
|  |  | |  |  | |  | |  | | |  | | |  |
|  |  | |  |  | |  | |  | | |  | | |  |
|  |  | |  |  | |  | |  | | |  | | |  |
|  |  | |  |  | |  | |  | | |  | | |  |
| Model 11: TotalSeed ~ Circumference + AxisWidth + CWSeedNo | | | | | |  | |  | | |  | | |  |
| Variable x | Variable y_1_ | | Variable y_2_ | Variable y_3_ | | Estimate | | SE | | | *t-*value | | | *p-*value |
| TotalSeed | Circumference | |  |  | | -360.0566 | | 134.0335 | | | -2.686 | | | 0.0187 |
| TotalSeed | AxisWidth | |  |  | | -349.9387 | | 140.6153 | | | -2.489 | | | 0.0272 |
| TotalSeed | CWSeedNo | |  |  | | -248.2925 | | 93.4778 | | | -2.656 | | | 0.0198 |
| TotalSeed | Circumference | | AxisWidth |  | | 17.6053 | | 6.9126 | | | 2.547 | | | 0.0243 |
| TotalSeed | Circumference | | CWSeedNo |  | | 13.0236 | | 4.7198 | | | 2.759 | | | 0.0162 |
| TotalSeed | AxisWidth | | CWSeedNo |  | | 11.6776 | | 4.7583 | | | 2.454 | | | 0.029 |
| TotalSeed | Circumference | | AxisWidth | CWSeedNo | | -0.5966 | | 0.2351 | | | -2.537 | | | 0.0248 |
|  |  | |  |  | |  | |  | | |  | | |  |
| Model Fit Statistics | | |  |  | |  | |  | | |  | | |  |
| Multiple R^2^ | 0.635 | |  |  | |  | |  | | |  | | |  |
| Adjusted R^2^ | | 0.438 |  | |  | |  | | |  | | |  | |
| *F-*statistic | 3.23 | |  |  | |  | |  | | |  | | |  |
| df | 7, 13 | |  |  | |  | |  | | |  | | |  |
| *p-*value | 3.23 | |  |  | |  | |  | | |  | | |  |
|  |  | |  |  | |  | |  | | |  | | |  |
|  |  | |  |  | |  | |  | | |  | | |  |
|  |  | |  |  | |  | |  | | |  | | |  |
|  |  | |  |  | |  | |  | | |  | | |  |
|  |  | |  |  | |  | |  | | |  | | |  |
|  |  | |  |  | |  | |  | | |  | | |  |
|  |  | |  |  | |  | |  | | |  | | |  |
|  |  | |  |  | |  | |  | | |  | | |  |
|  |  | |  |  | |  | |  | | |  | | |  |
|  |  | |  |  | |  | |  | | |  | | |  |
|  |  | |  |  | |  | |  | | |  | | |  |
|  |  | |  |  | |  | |  | | |  | | |  |
|  |  | |  |  | |  | |  | | |  | | |  |
|  |  | |  |  | |  | |  | | |  | | |  |
| Model 12: TotalSeed ~ AxisLength + AxisWidth + CWSeedNo + CCWSeedNo | | | | | | | |  | | |  | | |  |
| Variable x | Variable y_1_ | | Variable y_2_ | Variable y_3_ | | Estimate | | SE | | | *t-*value | | | *p-*value |
| TotalSeed | AxisLength | |  |  | | -2169.4808 | | 2187.1779 | | | -0.992 | | | 0.367 |
| TotalSeed | AxisWidth | |  |  | | -3603.9116 | | 3973.8094 | | | -0.907 | | | 0.406 |
| TotalSeed | CWSeedNo | |  |  | | -3357.86 | | 3314.7522 | | | -1.013 | | | 0.358 |
| TotalSeed | CCWSeedNo | |  |  | | -2848.6708 | | 2949.8792 | | | -0.966 | | | 0.379 |
| TotalSeed | AxisLength | | AxisWidth |  | | 84.2658 | | 97.2725 | | | 0.866 | | | 0.426 |
| TotalSeed | AxisLength | | CWSeedNo |  | | 81.2554 | | 80.0953 | | | 1.014 | | | 0.357 |
| TotalSeed | AxisWidth | | CWSeedNo |  | | 139.1585 | | 152.0983 | | | 0.915 | | | 0.402 |
| TotalSeed | AxisLength | | CCWSeedNo |  | | 67.9703 | | 71.794 | | | 0.947 | | | 0.387 |
| TotalSeed | AxisWidth | | CCWSeedNo |  | | 114.3304 | | 133.1391 | | | 0.859 | | | 0.43 |
| TotalSeed | CWSeedNo | | CCWSeedNo |  | | 109.1038 | | 112.2951 | | | 0.972 | | | 0.376 |
| TotalSeed | AxisLength | | AxisWidth | CWSeedNo | | -3.2753 | | 3.6592 | | | -0.895 | | | 0.412 |
| TotalSeed | AxisLength | | AxisWidth | CCWSeedNo | | -2.6256 | | 3.2282 | | | -0.813 | | | 0.453 |
| TotalSeed | AxisLength | | CWSeedNo | CCWSeedNo | | -2.6145 | | 2.6917 | | | -0.971 | | | 0.376 |
| TotalSeed | AxisWidth | | CWSeedNo | CCWSeedNo | | -4.5505 | | 5.2269 | | | -0.871 | | | 0.424 |
|  |  | |  |  | |  | |  | | |  | | |  |
| Model Fit Statistics | | |  |  | |  | |  | | |  | | |  |
| Multiple R^2^ | 0.792 | |  |  | |  | |  | | |  | | |  |
| Adjusted R^2^ | | 0.168 |  | |  | |  | | |  | | |  | |
| *F-*statistic | | 1.269 |  | |  | |  | | |  | | |  | |
| df | 15, 5 | |  |  | |  | |  | | |  | | |  |
| *p-*value | 0.4256 | |  |  | |  | |  | | |  | | |  |
|  |  | |  |  | |  | |  | | |  | | |  |
|  |  | |  |  | |  | |  | | |  | | |  |
|  |  | |  |  | |  | |  | | |  | | |  |
|  |  | |  |  | |  | |  | | |  | | |  |
|  |  | |  |  | |  | |  | | |  | | |  |
|  |  | |  |  | |  | |  | | |  | | |  |
|  |  | |  |  | |  | |  | | |  | | |  |
| Model 13: TotalSeed ~ Circumference + AxisLength + CWSeedNo + CWNo | | | | | | | |  | | |  | | |  |
| Variable x | Variable y_1_ | | Variable y_2_ | Variable y_3_ | | Estimate | | SE | | | *t-*value | | | *p-*value |
| TotalSeed | Circumference | |  |  | | 2777.00 | | 3975.00 | | | 0.698 | | | 0.516 |
| TotalSeed | AxisLength | |  |  | | 1420.00 | | 1882.00 | | | 0.755 | | | 0.485 |
| TotalSeed | CWSeedNo | |  |  | | 1687.00 | | 2831.00 | | | 0.596 | | | 0.577 |
| TotalSeed | CWNo | |  |  | | 2621.00 | | 4241.00 | | | 0.618 | | | 0.564 |
| TotalSeed | Circumference | | AxisLength |  | | -67.33 | | 91.07 | | | -0.739 | | | 0.493 |
| TotalSeed | Circumference | | CWSeedNo |  | | -78.91 | | 134.80 | | | -0.585 | | | 0.584 |
| TotalSeed | AxisLength | | CWSeedNo |  | | -39.28 | | 67.33 | | | -0.583 | | | 0.585 |
| TotalSeed | Circumference | | CWNo |  | | -119.90 | | 200.50 | | | -0.598 | | | 0.576 |
| TotalSeed | AxisLength | | CWNo |  | | -66.74 | | 106.80 | | | -0.625 | | | 0.559 |
| TotalSeed | CWSeedNo | | CWNo |  | | -70.69 | | 151.80 | | | -0.466 | | | 0.661 |
| TotalSeed | Circumference | | AxisLength | CWSeedNo | | 1.80 | | 3.17 | | | 0.567 | | | 0.595 |
| TotalSeed | Circumference | | AxisLength | CWNo | | 2.97 | | 4.97 | | | 0.597 | | | 0.577 |
| TotalSeed | Circumference | | CWSeedNo | CWNo | | 3.02 | | 6.88 | | | 0.439 | | | 0.679 |
| TotalSeed | AxisLength | | CWSeedNo | CWNo | | 1.66 | | 3.95 | | | 0.419 | | | 0.692 |
|  |  | |  |  | |  | |  | | |  | | |  |
| Model Fit Statistics | | |  |  | |  | |  | | |  | | |  |
| Multiple R^2^ | 0.9075 | |  |  | |  | |  | | |  | | |  |
| Adjusted R^2^ | | 0.63 |  | |  | |  | | |  | | |  | |
| *F-*statistic | | 3.271 |  | |  | |  | | |  | | |  | |
| df | 15, 5 | |  |  | |  | |  | | |  | | |  |
| *p-*value | 0.09814 | |  |  | |  | |  | | |  | | |  |
|  |  | |  |  | |  | |  | | |  | | |  |
|  |  | |  |  | |  | |  | | |  | | |  |
|  |  | |  |  | |  | |  | | |  | | |  |
|  |  | |  |  | |  | |  | | |  | | |  |
|  |  | |  |  | |  | |  | | |  | | |  |
|  |  | |  |  | |  | |  | | |  | | |  |
|  |  | |  |  | |  | |  | | |  | | |  |
| Model 14: CWNo ~ CWDeg + CCWDeg | | | | | | | |  | | |  | | |  |
| Variable x | Variable y_1_ | | Variable y_2_ | Variable y_3_ | | Estimate | | SE | | | *t-*value | | | *p-*value |
| CWNo | CWDeg | |  |  | | -0.000606 | | 0.03276 | | | -0.018 | | | 0.985 |
| CWNo | CCWDeg | |  |  | | 0.00914 | | 0.03152 | | | 0.29 | | | 0.775 |
| CWNo | CWDeg | | CCWDeg |  | | -0.0000283 | | 0.00009156 | | | -0.309 | | | 0.761 |
|  |  | |  |  | |  | |  | | |  | | |  |
| Model Fit Statistics | | | Multiple R^2^ | Adjusted R^2^ | | *F-*statistic | | df | | | *p-*value | | |  |
| Multiple R^2^ | 0.179 | |  |  | |  | |  | | |  | | |  |
| Adjusted R^2^ | | 0.0341 |  | |  | |  | | |  | | |  | |
| *F-*statistic | 1.235 | |  |  | |  | |  | | |  | | |  |
| df | 3, 17 | |  |  | |  | |  | | |  | | |  |
| *p-*value | 0.3277 | |  |  | |  | |  | | |  | | |  |
|  |  | |  |  | |  | |  | | |  | | |  |
|  |  | |  |  | |  | |  | | |  | | |  |
| Model 15: CWNo ~ CWSeedNo + CCWSeedNo | | | |  | |  | |  | | |  | | |  |
| Variable x | Variable y_1_ | | Variable y_2_ | Variable y_3_ | | Estimate | | SE | | | *t-*value | | | *p-*value |
| CWNo | CWSeedNo | |  |  | | 0.07364 | | 0.76201 | | | 0.097 | | | 0.924 |
| CWNo | CCWSeedNo | |  |  | | 0.46815 | | 0.74539 | | | 0.628 | | | 0.538 |
| CWNo | CWSeedNo | | CCWSeedNo |  | | -0.01472 | | 0.02527 | | | -0.583 | | | 0.568 |
|  |  | |  |  | |  | |  | | |  | | |  |
| Model Fit Statistics | | | Multiple R^2^ | Adjusted R^2^ | | *F-*statistic | | df | | | *p-*value | | |  |
| Multiple R^2^ | 0.4296 | |  |  | |  | |  | | |  | | |  |
| Adjusted R^2^ | | 0.329 |  | |  | |  | | |  | | |  | |
| *F-*statistic | 4.269 | |  |  | |  | |  | | |  | | |  |
| df | 3, 17 | |  |  | |  | |  | | |  | | |  |
| *p-*value | 0.02029 | |  |  | |  | |  | | |  | | |  |
|  |  | |  |  | |  | |  | | |  | | |  |
|  |  | |  |  | |  | |  | | |  | | |  |
|  |  | |  |  | |  | |  | | |  | | |  |
|  |  | |  |  | |  | |  | | |  | | |  |
| Model 16: CWNo ~ CWDeg + CWSeedNo | | | | | |  | |  | | |  | | |  |
| Variable x | Variable y_1_ | | Variable y_2_ | Variable y_3_ | | Estimate | | SE | | | *t-*value | | | *p-*value |
| CWNo | CWDeg | |  |  | | -0.0159826 | | 0.0256505 | | | -0.623 | | | 0.5415 |
| CWNo | CWSeedNo | |  |  | | -0.7341249 | | 0.2997343 | | | -2.449 | | | 0.0255 |
| CWNo | CWDeg | | CWSeedNo |  | | 0.000638 | | 0.0006859 | | | 0.93 | | | 0.3653 |
|  |  | |  |  | |  | |  | | |  | | |  |
| Model Fit Statistics | | |  |  | |  | |  | | |  | | |  |
| Multiple R^2^ | 0.4687 | |  |  | |  | |  | | |  | | |  |
| Adjusted R^2^ | | 0.375 |  | |  | |  | | |  | | |  | |
| *F-*statistic | 4.999 | |  |  | |  | |  | | |  | | |  |
| df | 3, 17 | |  |  | |  | |  | | |  | | |  |
| *p-*value | 0.01149 | |  |  | |  | |  | | |  | | |  |
|  |  | |  |  | |  | |  | | |  | | |  |
|  |  | |  |  | |  | |  | | |  | | |  |
| Model 17: CWNo ~ Circumference + Length + Width | | | | | |  | |  | | |  | | |  |
| Variable x | Variable y_1_ | | Variable y_2_ | Variable y_3_ | | Estimate | | SE | | | *t-*value | | | *p-*value |
| CWNo | Circumference | |  |  | | -5.02E+00 | | 1.41E+01 | | | -0.356 | | | 0.727 |
| CWNo | Length | |  |  | | 1.30E+00 | | 2.83E+00 | | | 0.458 | | | 0.654 |
| CWNo | Width | |  |  | | 8.52E-01 | | 4.36E+00 | | | 0.196 | | | 0.848 |
| CWNo | Circumference | | Length |  | | 2.35E-02 | | 2.12E-01 | | | 0.111 | | | 0.913 |
| CWNo | Circumference | | Width |  | | 2.48E-02 | | 1.91E-01 | | | 0.13 | | | 0.899 |
| CWNo | Length | | Width |  | | -4.61E-02 | | 6.14E-02 | | | -0.75 | | | 0.467 |
| CWNo | Circumference | | Length | Width | | 9.27E-04 | | 2.59E-03 | | | 0.358 | | | 0.726 |
|  |  | |  |  | |  | |  | | |  | | |  |
| Model Fit Statistics | | |  |  | |  | |  | | |  | | |  |
| Multiple R^2^ | 0.5243 | |  |  | |  | |  | | |  | | |  |
| Adjusted R^2^ | | 0.2682 |  | |  | |  | | |  | | |  | |
| *F-*statistic | 2.047 | |  |  | |  | |  | | |  | | |  |
| df | 7, 13 | |  |  | |  | |  | | |  | | |  |
| *p-*value | 0.1256 | |  |  | |  | |  | | |  | | |  |
| Model 18: CWNo ~ Circumference + OntoDir + AxisLength + TotalSeed | | | | | | | |  | | |  | | |  |
| Variable x | Variable y_1_ | | Variable y_2_ | Variable y_3_ | | Estimate | | SE | | | *t-*value | | | *p-*value |
| CWNo | Circumference | |  |  | | -5.86E+01 | | 1.96E+01 | | | -2.994 | | | 0.0303 |
| CWNo | OntoDir | |  |  | | -4.41E+03 | | 1.41E+03 | | | -3.123 | | | 0.0262 |
| CWNo | AxisLength | |  |  | | -3.73E+01 | | 1.26E+01 | | | -2.957 | | | 0.0316 |
| CWNo | TotalSeed | |  |  | | -3.35E+00 | | 1.09E+00 | | | -3.066 | | | 0.0279 |
| CWNo | Circumference | | OntoDir |  | | 2.01E+02 | | 6.13E+01 | | | 3.27 | | | 0.0222 |
| CWNo | Circumference | | AxisLength |  | | 1.72E+00 | | 6.05E-01 | | | 2.84 | | | 0.0362 |
| CWNo | OntoDir | | AxisLength |  | | 1.08E+02 | | 3.02E+01 | | | 3.582 | | | 0.0158 |
| CWNo | Circumference | | TotalSeed |  | | 1.57E-01 | | 5.24E-02 | | | 2.987 | | | 0.0306 |
| CWNo | OntoDir | | TotalSeed |  | | 1.56E+01 | | 5.88E+00 | | | 2.646 | | | 0.0456 |
| CWNo | AxisLength | | TotalSeed |  | | 9.62E-02 | | 3.38E-02 | | | 2.841 | | | 0.0362 |
| CWNo | Circumference | | OntoDir | AxisLength | | -4.91E+00 | | 1.40E+00 | | | -3.516 | | | 0.017 |
| CWNo | Circumference | | OntoDir | TotalSeed | | -7.36E-01 | | 2.58E-01 | | | -2.849 | | | 0.0358 |
| CWNo | Circumference | | AxisLength | TotalSeed | | -4.49E-03 | | 1.62E-03 | | | -2.772 | | | 0.0393 |
| CWNo | OntoDir | | AxisLength | TotalSeed | | -3.82E-01 | | 1.29E-01 | | | -2.974 | | | 0.031 |
|  |  | |  |  | |  | |  | | |  | | |  |
| Model Fit Statistics | | |  |  | |  | |  | | |  | | |  |
| Multiple R^2^ | 0.979 | |  |  | |  | |  | | |  | | |  |
| Adjusted R^2^ | | 0.914 |  | |  | |  | | |  | | |  | |
| *F-*statistic | 15.3 | |  |  | |  | |  | | |  | | |  |
| df | 15, 5 | |  |  | |  | |  | | |  | | |  |
| *p-*value | 0.00353 | |  |  | |  | |  | | |  | | |  |
|  |  | |  |  | |  | |  | | |  | | |  |
|  |  | |  |  | |  | |  | | |  | | |  |
|  |  | |  |  | |  | |  | | |  | | |  |
|  |  | |  |  | |  | |  | | |  | | |  |
|  |  | |  |  | |  | |  | | |  | | |  |
|  |  | |  |  | |  | |  | | |  | | |  |
|  |  | |  |  | |  | |  | | |  | | |  |
| Model 19: CCWNo ~ CWDeg + CCWDeg | | | | | |  | |  | | |  | | |  |
| Variable x | Variable y_1_ | | Variable y_2_ | Variable y_3_ | | Estimate | | SE | | | *t-*value | | | *p-*value |
| CCWNo | CWDeg | |  |  | | 7.23E-03 | | 3.30E-02 | | | 0.219 | | | 0.829 |
| CCWNo | CCWDeg | |  |  | | 7.78E-03 | | 3.17E-02 | | | 0.245 | | | 0.809 |
| CCWNo | CWDeg | | CCWDeg |  | | -4.24E-06 | | 9.21E-05 | | | -0.046 | | | 0.964 |
|  |  | |  |  | |  | |  | | |  | | |  |
| Model Fit Statistics | | |  |  | |  | |  | | |  | | |  |
| Multiple R^2^ | 0.08247 | |  |  | |  | |  | | |  | | |  |
| Adjusted R^2^ | | -0.07945 |  | |  | |  | | |  | | |  | |
| *F-*statistic | 0.5093 | |  |  | |  | |  | | |  | | |  |
| df | 3, 17 | |  |  | |  | |  | | |  | | |  |
| *p-*value | 0.6811 | |  |  | |  | |  | | |  | | |  |
|  |  | |  |  | |  | |  | | |  | | |  |
|  |  | |  |  | |  | |  | | |  | | |  |
| Model 20: CCWNo ~ CWSeedNo + CCWSeedNo | | | | | |  | |  | | |  | | |  |
| Variable x | Variable y_1_ | | Variable y_2_ | Variable y_3_ | | Estimate | | SE | | | *t-*value | | | *p-*value |
| CCWNo | CWSeedNo | |  |  | | -0.076334 | | 0.907046 | | | -0.084 | | | 0.934 |
| CCWNo | CCWSeedNo | |  |  | | -0.236285 | | 0.887257 | | | -0.266 | | | 0.793 |
| CCWNo | CWSeedNo | | CCWSeedNo |  | | 0.008746 | | 0.030083 | | | 0.291 | | | 0.775 |
|  |  | |  |  | |  | |  | | |  | | |  |
| Model Fit Statistics | | |  |  | |  | |  | | |  | | |  |
| Multiple R^2^ | 0.1082 | |  |  | |  | |  | | |  | | |  |
| Adjusted R^2^ | | -0.04914 |  | |  | |  | | |  | | |  | |
| *F-*statistic | 0.6877 | |  |  | |  | |  | | |  | | |  |
| df | 3, 17 | |  |  | |  | |  | | |  | | |  |
| *p-*value | 0.5718 | |  |  | |  | |  | | |  | | |  |
|  |  | |  |  | |  | |  | | |  | | |  |
|  |  | |  |  | |  | |  | | |  | | |  |
|  |  | |  |  | |  | |  | | |  | | |  |
|  |  | |  |  | |  | |  | | |  | | |  |
| Model 21: CCWNo ~ CWDeg + CWSeedNo | | | | | |  | |  | | |  | | |  |
| Variable x | Variable y_1_ | | Variable y_2_ | Variable y_3_ | | Estimate | | SE | | | *t-*value | | | *p-*value |
| CCWNo | CWDeg | |  |  | | -0.0003557 | | 0.0310328 | | | -0.011 | | | 0.991 |
| CCWNo | CWSeedNo | |  |  | | 0.3922168 | | 0.3626283 | | | 1.082 | | | 0.295 |
| CCWNo | CWDeg | | CWSeedNo |  | | -0.0002004 | | 0.0008298 | | | -0.242 | | | 0.812 |
|  |  | |  |  | |  | |  | | |  | | |  |
| Model Fit Statistics | | |  |  | |  | |  | | |  | | |  |
| Multiple R^2^ | 0.1419 | |  |  | |  | |  | | |  | | |  |
| Adjusted R^2^ | | -0.009541 |  | |  | |  | | |  | | |  | |
| *F-*statistic | 0.937 | |  |  | |  | |  | | |  | | |  |
| df | 3, 17 | |  |  | |  | |  | | |  | | |  |
| *p-*value | 0.4445 | |  |  | |  | |  | | |  | | |  |
|  |  | |  |  | |  | |  | | |  | | |  |
|  |  | |  |  | |  | |  | | |  | | |  |
| Model 22: CCWNo ~ Circumference + Length + Width | | | | | |  | |  | | |  | | |  |
| Variable x | Variable y_1_ | | Variable y_2_ | Variable y_3_ | | Estimate | | SE | | | *t-*value | | | *p-*value |
| CCWNo | Circumference | |  |  | | 15.42 | | 17.62 | | | 0.875 | | | 0.397 |
| CCWNo | Length | |  |  | | 0.97 | | 3.54 | | | 0.273 | | | 0.79 |
| CCWNo | Width | |  |  | | 2.01 | | 5.45 | | | 0.369 | | | 0.718 |
| CCWNo | Circumference | | Length |  | | -0.17 | | 0.27 | | | -0.648 | | | 0.528 |
| CCWNo | Circumference | | Width |  | | -0.20 | | 0.24 | | | -0.853 | | | 0.409 |
| CCWNo | Length | | Width |  | | 0.00 | | 0.08 | | | -0.032 | | | 0.975 |
| CCWNo | Circumference | | Length | Width | | 0.00 | | 0.00 | | | 0.578 | | | 0.573 |
|  |  | |  |  | |  | |  | | |  | | |  |
| Model Fit Statistics | | |  |  | |  | |  | | |  | | |  |
| Multiple R^2^ | 0.1788 | |  |  | |  | |  | | |  | | |  |
| Adjusted R^2^ | | -0.2633 |  | |  | |  | | |  | | |  | |
| *F-*statistic | 0.4045 | |  |  | |  | |  | | |  | | |  |
| df | 7, 13 | |  |  | |  | |  | | |  | | |  |
| *p-*value | 0.8829 | |  |  | |  | |  | | |  | | |  |
| Model 23: CCWNo ~ Circumference + OntoDir | | | | | |  | |  | | |  | | |  |
| Variable x | Variable y_1_ | | Variable y_2_ | Variable y_3_ | | Estimate | | SE | | | *t-*value | | | *p-*value |
| CCWNo | Circumference | |  |  | | 0.3429 | | 0.3738 | | | 0.917 | | | 0.3718 |
| CCWNo | OntoDir | |  |  | | 26.0654 | | 13.8093 | | | 1.888 | | | 0.0763 |
| CCWNo | Circumference | | OntoDir |  | | -1.2407 | | 0.6836 | | | -1.815 | | | 0.0872 |
|  |  | |  |  | |  | |  | | |  | | |  |
| Model Fit Statistics | | |  |  | |  | |  | | |  | | |  |
| Multiple R^2^ | 0.18 | |  |  | |  | |  | | |  | | |  |
| Adjusted R^2^ | | 0.0356 |  | |  | |  | | |  | | |  | |
| *F-*statistic | 1.25 | |  |  | |  | |  | | |  | | |  |
| df | 3, 17 | |  |  | |  | |  | | |  | | |  |
| *p-*value | 0.324 | |  |  | |  | |  | | |  | | |  |
|  |  | |  |  | |  | |  | | |  | | |  |
|  |  | |  |  | |  | |  | | |  | | |  |
| Model 24: CWDeg ~ CWNo + CCWNo | | | | | |  | |  | | |  | | |  |
| Variable x | Variable y_1_ | | Variable y_2_ | Variable y_3_ | | Estimate | | SE | | | *t-*value | | | *p-*value |
| CWDeg | CWNo | |  |  | | 174.69 | | 186.81 | | | 0.935 | | | 0.363 |
| CWDeg | CCWNo | |  |  | | 187.07 | | 184.57 | | | 1.014 | | | 0.325 |
| CWDeg | CWNo | | CCWNo |  | | -14.21 | | 13.72 | | | -1.036 | | | 0.315 |
|  |  | |  |  | |  | |  | | |  | | |  |
| Model Fit Statistics | | |  |  | |  | |  | | |  | | |  |
| Multiple R^2^ | 0.229 | |  |  | |  | |  | | |  | | |  |
| Adjusted R^2^ | | 0.0929 |  | |  | |  | | |  | | |  | |
| *F-*statistic | 1.68 | |  |  | |  | |  | | |  | | |  |
| df | 3, 17 | |  |  | |  | |  | | |  | | |  |
| *p-*value | 0.208 | |  |  | |  | |  | | |  | | |  |
|  |  | |  |  | |  | |  | | |  | | |  |
|  |  | |  |  | |  | |  | | |  | | |  |
|  |  | |  |  | |  | |  | | |  | | |  |
|  |  | |  |  | |  | |  | | |  | | |  |
| Model 25: CWDeg ~ CWSeedNo + CCWSeedNo | | | | | |  | |  | | |  | | |  |
| Variable x | Variable y_1_ | | Variable y_2_ | Variable y_3_ | | Estimate | | SE | | | *t-*value | | | *p-*value |
| CWDeg | CWSeedNo | |  |  | | 25.5632 | | 24.1188 | | | 1.06 | | | 0.304 |
| CWDeg | CCWSeedNo | |  |  | | 7.1378 | | 23.5926 | | | 0.303 | | | 0.766 |
| CWDeg | CWSeedNo | | CCWSeedNo |  | | -0.2182 | | 0.7999 | | | -0.273 | | | 0.788 |
|  |  | |  |  | |  | |  | | |  | | |  |
| Model Fit Statistics | | |  |  | |  | |  | | |  | | |  |
| Multiple R^2^ | 0.644 | |  |  | |  | |  | | |  | | |  |
| Adjusted R^2^ | | 0.582 |  | |  | |  | | |  | | |  | |
| *F-*statistic | 10.3 | |  |  | |  | |  | | |  | | |  |
| df | 3, 17 | |  |  | |  | |  | | |  | | |  |
| *p-*value | 0.000431 | |  |  | |  | |  | | |  | | |  |
|  |  | |  |  | |  | |  | | |  | | |  |
|  |  | |  |  | |  | |  | | |  | | |  |
| Model 26: CCWDeg ~ CWNo + CCWNo | | | | | | | |  | | |  | | |  |
| Variable x | Variable y_1_ | | Variable y_2_ | Variable y_3_ | | Estimate | | SE | | | *t-*value | | | *p-*value |
| CCWDeg | CWNo | |  |  | | -117.194 | | 199.21 | | | -0.588 | | | 0.564 |
| CCWDeg | CCWNo | |  |  | | -113.885 | | 196.822 | | | -0.579 | | | 0.57 |
| CCWDeg | CWNo | | CCWNo |  | | 9.525 | | 14.627 | | | 0.651 | | | 0.524 |
|  |  | |  |  | |  | |  | | |  | | |  |
| Model Fit Statistics | | |  |  | |  | |  | | |  | | |  |
| Multiple R^2^ | 0.124 | |  |  | |  | |  | | |  | | |  |
| Adjusted R^2^ | | -0.0304 |  | |  | |  | | |  | | |  | |
| *F-*statistic | 0.803 | |  |  | |  | |  | | |  | | |  |
| df | 3, 17 | |  |  | |  | |  | | |  | | |  |
| *p-*value | 0.509 | |  |  | |  | |  | | |  | | |  |
|  |  | |  |  | |  | |  | | |  | | |  |
|  |  | |  |  | |  | |  | | |  | | |  |
|  |  | |  |  | |  | |  | | |  | | |  |
|  |  | |  |  | |  | |  | | |  | | |  |
| Model 27: CCWDeg ~ CWSeedNo + CCWSeedNo | | | | | |  | |  | | |  | | |  |
| Variable x | Variable y_1_ | | Variable y_2_ | Variable y_3_ | | Estimate | | SE | | | *t-*value | | | *p-*value |
| CCWDeg | CWSeedNo | |  |  | | 13.032 | | 28.396 | | | 0.459 | | | 0.652 |
| CCWDeg | CCWSeedNo | |  |  | | 31.37 | | 27.7765 | | | 1.129 | | | 0.274 |
| CCWDeg | CWSeedNo | | CCWSeedNo |  | | -0.5109 | | 0.9418 | | | -0.542 | | | 0.595 |
|  |  | |  |  | |  | |  | | |  | | |  |
| Model Fit Statistics | | |  |  | |  | |  | | |  | | |  |
| Multiple R^2^ | 0.508 | |  |  | |  | |  | | |  | | |  |
| Adjusted R^2^ | | 0.421 |  | |  | |  | | |  | | |  | |
| *F-*statistic | 5.85 | |  |  | |  | |  | | |  | | |  |
| df | 3, 17 | |  |  | |  | |  | | |  | | |  |
| *p-*value | 0.00621 | |  |  | |  | |  | | |  | | |  |
|  |  | |  |  | |  | |  | | |  | | |  |
|  |  | |  |  | |  | |  | | |  | | |  |
|  |  | |  |  | |  | |  | | |  | | |  |
|  |  | |  |  | |  | |  | | |  | | |  |
|  |  | |  |  | |  | |  | | |  | | |  |
|  |  | |  |  | |  | |  | | |  | | |  |
|  |  | |  |  | |  | |  | | |  | | |  |
|  |  | |  |  | |  | |  | | |  | | |  |
|  |  | |  |  | |  | |  | | |  | | |  |
|  |  | |  |  | |  | |  | | |  | | |  |
|  |  | |  |  | |  | |  | | |  | | |  |
|  |  | |  |  | |  | |  | | |  | | |  |
|  |  | |  |  | |  | |  | | |  | | |  |
|  |  | |  |  | |  | |  | | |  | | |  |
|  |  | |  |  | |  | |  | | |  | | |  |
|  |  | |  |  | |  | |  | | |  | | |  |
|  |  | |  |  | |  | |  | | |  | | |  |
|  |  | |  |  | |  | |  | | |  | | |  |
| Model 28: CWDeg ~ AxisLength + AxisWidth + Circumference + Width | | | | | | | |  | | |  | | |  |
| Variable x | Variable y_1_ | | Variable y_2_ | Variable y_3_ | | Estimate | | SE | | | *t-*value | | | *p-*value |
| CWDeg | AxisLength | |  |  | | 2.19E+03 | | 1.34E+03 | | | 1.639 | | | 0.16214 |
| CWDeg | AxisWidth | |  |  | | -1.28E+04 | | 5.07E+03 | | | -2.528 | | | 0.05064 |
| CWDeg | Circumference | |  |  | | 7.07E+03 | | 3.86E+03 | | | 1.829 | | | 0.12692 |
| CWDeg | Width | |  |  | | -7.65E+03 | | 2.01E+03 | | | -3.799 | | | 0.01264 |
| CWDeg | AxisLength | | AxisWidth |  | | 2.03E+01 | | 7.91E+01 | | | 0.257 | | | 0.80734 |
| CWDeg | AxisLength | | Circumference |  | | -5.31E+02 | | 1.05E+02 | | | -5.049 | | | 0.00394 |
| CWDeg | AxisWidth | | Circumference |  | | -8.00E+01 | | 2.18E+02 | | | -0.366 | | | 0.72907 |
| CWDeg | AxisLength | | Width |  | | 1.06E+02 | | 3.21E+01 | | | 3.302 | | | 0.02142 |
| CWDeg | AxisWidth | | Width |  | | 4.05E+02 | | 1.07E+02 | | | 3.774 | | | 0.01298 |
| CWDeg | Circumference | | AxisWidth |  | | 1.44E+02 | | 7.89E+01 | | | 1.82 | | | 0.12844 |
| CWDeg | AxisLength | | AxisWidth | Circumference | | 1.78E+01 | | 4.80E+00 | | | 3.705 | | | 0.01393 |
| CWDeg | AxisLength | | AxisWidth | Width | | -6.11E+00 | | 1.82E+00 | | | -3.353 | | | 0.02026 |
| CWDeg | AxisLength | | Circumference | Width | | 9.92E-01 | | 1.32E+00 | | | 0.752 | | | 0.48609 |
| CWDeg | AxisWidth | | Circumference | Width | | -9.38E+00 | | 4.32E+00 | | | -2.172 | | | 0.08196 |
|  |  | |  |  | |  | |  | | |  | | |  |
| Model Fit Statistics | | |  |  | |  | |  | | |  | | |  |
| Multiple R^2^ | 0.964 | |  |  | |  | |  | | |  | | |  |
| Adjusted R^2^ | | 0.856 |  | |  | |  | | |  | | |  | |
| *F-*statistic | 8.91 | |  |  | |  | |  | | |  | | |  |
| df | 15, 5 | |  |  | |  | |  | | |  | | |  |
| *p-*value | 1.22E-02 | |  |  | |  | |  | | |  | | |  |
|  |  | |  |  | |  | |  | | |  | | |  |
|  |  | |  |  | |  | |  | | |  | | |  |
|  |  | |  |  | |  | |  | | |  | | |  |
|  |  | |  |  | |  | |  | | |  | | |  |
|  |  | |  |  | |  | |  | | |  | | |  |
|  |  | |  |  | |  | |  | | |  | | |  |
|  |  | |  |  | |  | |  | | |  | | |  |
| Model 29: CWSeedNo ~ CWNo + CCWNo | | | | | |  | |  | | |  | | |  |
| Variable x | Variable y_1_ | | Variable y_2_ | Variable y_3_ | | Estimate | | SE | | | *t-*value | | | *p-*value |
| CWSeedNo | CWNo | |  |  | | 11.2044 | | 6.2522 | | | 1.792 | | | 0.0909 |
| CWSeedNo | CCWNo | |  |  | | 12.147 | | 6.1772 | | | 1.966 | | | 0.0658 |
| CWSeedNo | CWNo | | CCWNo |  | | -0.9051 | | 0.4591 | | | -1.972 | | | 0.0652 |
|  |  | |  |  | |  | |  | | |  | | |  |
| Model Fit Statistics | | |  |  | |  | |  | | |  | | |  |
| Multiple R^2^ | 0.524 | |  |  | |  | |  | | |  | | |  |
| Adjusted R^2^ | | 0.439 |  | |  | |  | | |  | | |  | |
| *F-*statistic | 6.23 | |  |  | |  | |  | | |  | | |  |
| df | 3, 17 | |  |  | |  | |  | | |  | | |  |
| *p-*value | 0.00477 | |  |  | |  | |  | | |  | | |  |
|  |  | |  |  | |  | |  | | |  | | |  |
|  |  | |  |  | |  | |  | | |  | | |  |
| Model 30: CWSeedNo ~ CWDeg + CCWDeg | | | | | |  | |  | | |  | | |  |
| Variable x | Variable y_1_ | | Variable y_2_ | Variable y_3_ | | Estimate | | SE | | | *t-*value | | | *p-*value |
| CWSeedNo | CWDeg | |  |  | | 0.04 | | 0.04 | | | 1.069 | | | 0.3 |
| CWSeedNo | CCWDeg | |  |  | | 0.00 | | 0.04 | | | 0.136 | | | 0.894 |
| CWSeedNo | CWDeg | | CCWDeg |  | | 0.00 | | 0.00 | | | -0.152 | | | 0.881 |
|  |  | |  |  | |  | |  | | |  | | |  |
| Model Fit Statistics | | |  |  | |  | |  | | |  | | |  |
| Multiple R^2^ | 0.643 | |  |  | |  | |  | | |  | | |  |
| Adjusted R^2^ | | 0.58 |  | |  | |  | | |  | | |  | |
| *F-*statistic | 10.2 | |  |  | |  | |  | | |  | | |  |
| df | 3, 17 | |  |  | |  | |  | | |  | | |  |
| *p-*value | 0.00045 | |  |  | |  | |  | | |  | | |  |
|  |  | |  |  | |  | |  | | |  | | |  |
|  |  | |  |  | |  | |  | | |  | | |  |
|  |  | |  |  | |  | |  | | |  | | |  |
|  |  | |  |  | |  | |  | | |  | | |  |
| Model 31: CCWSeedNo ~ CWNo + CCWNo | | | | | |  | |  | | |  | | |  |
| Variable x | Variable y_1_ | | Variable y_2_ | Variable y_3_ | | Estimate | | SE | | | *t-*value | | | *p-*value |
| CCWSeedNo | CWNo | |  |  | | -4.2782 | | 8.2649 | | | -0.518 | | | 0.611 |
| CCWSeedNo | CCWNo | |  |  | | -4.5745 | | 8.1658 | | | -0.56 | | | 0.583 |
| CCWSeedNo | CWNo | | CCWNo |  | | 0.3507 | | 0.6068 | | | 0.578 | | | 0.571 |
|  |  | |  |  | |  | |  | | |  | | |  |
| Model Fit Statistics | | |  |  | |  | |  | | |  | | |  |
| Multiple R^2^ | 0.0905 | |  |  | |  | |  | | |  | | |  |
| Adjusted R^2^ | | -0.07 |  | |  | |  | | |  | | |  | |
| *F-*statistic | 0.564 | |  |  | |  | |  | | |  | | |  |
| df | 3, 17 | |  |  | |  | |  | | |  | | |  |
| *p-*value | 0.646 | |  |  | |  | |  | | |  | | |  |
|  |  | |  |  | |  | |  | | |  | | |  |
|  |  | |  |  | |  | |  | | |  | | |  |
| Model 32: CCWSeedNo ~ CWDeg + CCWDeg | | | | | | | |  | | |  | | |  |
| Variable x | Variable y_1_ | | Variable y_2_ | Variable y_3_ | | Estimate | | SE | | | *t-*value | | | *p-*value |
| CCWSeedNo | CWDeg | |  |  | | -0.0865012 | | 0.0360082 | | | -2.402 | | | 0.028 |
| CCWSeedNo | CCWDeg | |  |  | | -0.0555381 | | 0.0346413 | | | -1.603 | | | 0.1273 |
| CCWSeedNo | CWDeg | | CCWDeg |  | | 0.0002504 | | 0.0001006 | | | 2.488 | | | 0.0235 |
|  |  | |  |  | |  | |  | | |  | | |  |
| Model Fit Statistics | | |  |  | |  | |  | | |  | | |  |
| Multiple R^2^ | 0.628 | |  |  | |  | |  | | |  | | |  |
| Adjusted R^2^ | | 0.563 |  | |  | |  | | |  | | |  | |
| *F-*statistic | 9.57 | |  |  | |  | |  | | |  | | |  |
| df | 3, 17 | |  |  | |  | |  | | |  | | |  |
| *p-*value | 0.000625 | |  |  | |  | |  | | |  | | |  |
|  |  | |  |  | |  | |  | | |  | | |  |
|  |  | |  |  | |  | |  | | |  | | |  |
|  |  | |  |  | |  | |  | | |  | | |  |
|  |  | |  |  | |  | |  | | |  | | |  |
| Model 33: CCWSeedNo ~ AxisLength + AxisWidth + CCWDeg + CWDeg | | | | | | | |  | | |  | | |  |
| Variable x | Variable y_1_ | | Variable y_2_ | Variable y_3_ | | Estimate | | SE | | | *t-*value | | | *p-*value |
| CCWSeedNo | AxisLength | |  |  | | 6.25E+01 | | 7.18E+01 | | | 0.871 | | | 0.424 |
| CCWSeedNo | AxisWidth | |  |  | | 1.27E+02 | | 1.61E+02 | | | 0.786 | | | 0.467 |
| CCWSeedNo | CCWDeg | |  |  | | 5.91E+00 | | 7.77E+00 | | | 0.76 | | | 0.481 |
| CCWSeedNo | CWDeg | |  |  | | 6.00E+00 | | 6.00E+00 | | | 0.999 | | | 0.364 |
| CCWSeedNo | AxisLength | | AxisWidth |  | | -3.42E+00 | | 4.20E+00 | | | -0.814 | | | 0.453 |
| CCWSeedNo | AxisLength | | CCWDeg |  | | -1.60E-01 | | 2.10E-01 | | | -0.762 | | | 0.48 |
| CCWSeedNo | AxisWidth | | CCWDeg |  | | -3.44E-01 | | 4.62E-01 | | | -0.744 | | | 0.491 |
| CCWSeedNo | AxisLength | | CWDeg |  | | -1.78E-01 | | 1.71E-01 | | | -1.04 | | | 0.346 |
| CCWSeedNo | AxisWidth | | CWDeg |  | | -3.21E-01 | | 3.16E-01 | | | -1.016 | | | 0.356 |
| CCWSeedNo | CCWDeg | | CWDeg |  | | -1.67E-02 | | 1.70E-02 | | | -0.981 | | | 0.372 |
| CCWSeedNo | AxisLength | | AxisWidth | CCWDeg | | 8.90E-03 | | 1.23E-02 | | | 0.725 | | | 0.501 |
| CCWSeedNo | AxisLength | | AxisWidth | CWDeg | | 9.07E-03 | | 8.90E-03 | | | 1.019 | | | 0.355 |
| CCWSeedNo | AxisLength | | CCWDeg | CWDeg | | 4.79E-04 | | 4.92E-04 | | | 0.975 | | | 0.375 |
| CCWSeedNo | AxisWidth | | CCWDeg | CWDeg | | 9.04E-04 | | 9.02E-04 | | | 1.002 | | | 0.362 |
|  |  | |  |  | |  | |  | | |  | | |  |
| Model Fit Statistics | | |  |  | |  | |  | | |  | | |  |
| Multiple R^2^ | 0.854 | |  |  | |  | |  | | |  | | |  |
| Adjusted R^2^ | | 0.417 |  | |  | |  | | |  | | |  | |
| *F-*statistic | 1.95 | |  |  | |  | |  | | |  | | |  |
| df | 15, 5 | |  |  | |  | |  | | |  | | |  |
| *p-*value | 0.237 | |  |  | |  | |  | | |  | | |  |
|  |  | |  |  | |  | |  | | |  | | |  |
|  |  | |  |  | |  | |  | | |  | | |  |
|  |  | |  |  | |  | |  | | |  | | |  |
|  |  | |  |  | |  | |  | | |  | | |  |
|  |  | |  |  | |  | |  | | |  | | |  |
|  |  | |  |  | |  | |  | | |  | | |  |
|  |  | |  |  | |  | |  | | |  | | |  |
| Model 34: Circumference ~ AxisLength + AxisWidth | | | | | |  | |  | | |  | | |  |
| Variable x | Variable y_1_ | | Variable y_2_ | Variable y_3_ | | Estimate | | SE | | | *t-*value | | | *p-*value |
| Circumference | AxisLength | |  |  | | 0.8878 | | 0.3613 | | | 2.457 | | | 0.025 |
| Circumference | AxisWidth | |  |  | | 1.24533 | | 0.61617 | | | 2.021 | | | 0.0593 |
| Circumference | AxisLength | | AxisWidth |  | | -0.03221 | | 0.01711 | | | -1.883 | | | 0.077 |
|  |  | |  |  | |  | |  | | |  | | |  |
| Model Fit Statistics | | |  |  | |  | |  | | |  | | |  |
| Multiple R^2^ | 0.521 | |  |  | |  | |  | | |  | | |  |
| Adjusted R^2^ | | 0.436 |  | |  | |  | | |  | | |  | |
| *F-*statistic | 6.16 | |  |  | |  | |  | | |  | | |  |
| df | 3, 17 | |  |  | |  | |  | | |  | | |  |
| *p-*value | 0.00499 | |  |  | |  | |  | | |  | | |  |
